# What Magnitude Are Observed Non-Target Impacts from Weed Biocontrol?

**DOI:** 10.1371/journal.pone.0084847

**Published:** 2014-01-13

**Authors:** David Maxwell Suckling, René François Henri Sforza

**Affiliations:** 1 Biosecurity Group, The New Zealand Institute of Plant and Food Research Ltd, Christchurch, New Zealand; 2 Better Border Biosecurity, Christchurch, New Zealand; 3 European Biological Control Laboratory, USDA-ARS, Campus International de Baillarguet, Montferrier-sur-Lez, France; AgroParisTech, France

## Abstract

A systematic review focused by plant on non-target impacts from agents deliberately introduced for the biological control of weeds found significant non-target impacts to be rare. The magnitude of direct impact of 43 biocontrol agents on 140 non-target plants was retrospectively categorized using a risk management framework for ecological impacts of invasive species (minimal, minor, moderate, major, massive). The vast majority of agents introduced for classical biological control of weeds (>99% of 512 agents released) have had no known significant adverse effects on non-target plants thus far; major effects suppressing non-target plant populations could be expected to be detectable. Most direct non-target impacts on plants (91.6%) were categorized as minimal or minor in magnitude with no known adverse long-term impact on non-target plant populations, but a few cacti and thistles are affected at moderate (n = 3), major (n = 7) to massive (n = 1) scale. The largest direct impacts are from two agents (*Cactoblastis cactorum* on native cacti and *Rhinocyllus conicus* on native thistles), but these introductions would not be permitted today as more balanced attitudes exist to plant biodiversity, driven by both society and the scientific community. Our analysis shows (as far as is known), weed biological control agents have a biosafety track record of >99% of cases avoiding significant non-target impacts on plant populations. Some impacts could have been overlooked, but this seems unlikely to change the basic distribution of very limited adverse effects. Fewer non-target impacts can be expected in future because of improved science and incorporation of wider values. Failure to use biological control represents a significant opportunity cost from the certainty of ongoing adverse impacts from invasive weeds. It is recommended that a simple five-step scale be used to better communicate the risk of consequences from both action (classical biological control) and no action (ongoing impacts from invasive weeds).

## Introduction

Classical biological control of weeds involves the deliberate introduction of exotic organisms, or biological control agents, to manage weed problems in the invaded range. It offers an excellent and sustainable solution for invasive species [1], [2]. Exotic weeds in natural and managed ecosystems have long been targeted, starting with the cases of prickly pear (*Opuntia* sp.) in India (1863) [3], then Sri Lanka (1865) [4], and Australia (1912) [3], and lantana (*Lantana camara* L.) in Hawai'i in 1902 [1]. After some assessment of cost-benefit ratio, the process involves collecting exotic natural enemies to control a target invasive weed, usually followed by importing, rearing, testing, and release from quarantine for establishment. Host specificity tests are conducted in artificial and field conditions, and increasingly combined with ecological and molecular evaluations [5]. Deliberate release of natural enemies is subject to official approvals.

Reported benefits in USA from the major weed biocontrol programs in the 20^th^ century resulted in benefits (net of research costs) in excess of US$180M per annum [1], mainly from reduced ongoing costs of control using herbicides. Environmental benefits of replacing pesticides can be considered to be proportional in magnitude to market economy benefits [6]. In South Africa, biocontrol of weeds contributes to prevention of substantial losses to the economy over the scale of decades, where it prevents the loss of ecosystem services that contribute to human well-being, including water [7]. Highly favorable results have emerged from similar analyses in Australia [8], [9] and New Zealand [10]–[13]. Plant invasion continues to be a major concern nonetheless, with a lag phase of several decades, and new introductions further increasing net effects from the increase of global trade [14].

The increasing incidence and impact of invasive species is widely recognized as a major and increasing threat to food and fiber production, as well as ecosystem functioning [15], so it could be assumed that the need for classical biological control to mitigate costs is increasing. However, despite an increasing track record of success and specificity with improved scientific knowledge [16], classical biological control has been criticized in recent years, through emerging recognition of non-target impacts [17]–[20]. Solutions are clearly needed to better predict the risk of significant non-target impacts in order to gain societal, economic and environmental benefits, while mitigating risk. The obvious major risk is that of a host shift, or the preference for an adopted host (an indigenous species or a crop in the introduced environment), over the original host (the target). The threat is either to a native plant species at population level and to ecosystem function, or to a crop, by defoliation or seed predation resulting in a yield reduction [21], [22]. The risks arise in this scenario because the newly-released organisms are self-perpetuating and self-dispersing, but these traits also offer the benefit of self-sustaining management [23]. That said, comparisons of realized host range with the predicted host range [24], [25] can improve biosafety processes. However, a lack of agreement between retrospective laboratory tests and long-term field observations has led to the conclusion that very successful biological control agents without non-target impacts might never have been introduced because of overstated ecological risk in the laboratory [26]. Clearly both types of errors should be avoided from host range tests, where ecologically safe candidates are not released and the benefits of sustainable pest control are not realized, or, unsafe candidates are released and ecological damage results.

Until 2000, the frequency of cases of known non-target impacts from classical biological control of weeds was small, compared with the number of agents released [21], [22]. Fowler et al. [22] reported that 12 biocontrol agents released against weeds had been recorded attacking non-target plants. Six of these cases (1.5% of agents released) were not anticipated. However, while this insect-centric perspective appears to offer some support for classical biological control, it has also been noted that release of thistle seed weevil *Rhinocyllus conicus* (Col. Curculionidae) in North America has led to about half of the (previously) recorded non-target impacts [27]. The release was undertaken during an era when rangeland management of economically important thistles was overriding. Crops were highly valued compared to natural values of indigenous North American thistles in the 1960s. As a consequence, incorporation of ecological considerations was limited [28]. Interestingly, there is no evidence of non-target impacts from plant pathogens thus far [29], [30], but it may be too early to tell whether the organisms chosen are more host specific and therefore have a lower risk profile, since there have been far fewer introductions of plan pathogens so far. This field appears to offer good opportunity to avoid the mistakes of the past.

No overview of weed biological control studies has yet evaluated the of adverse non-target impact of all agents once released, separating effects reducing plant populations at ecological scale from effects which don't have such implications. In order to use an existing framework for such study, we followed Parker et al. [15] who suggested that the impact of an invader can be measured at five levels: (1) genetic effects, (2) effects on individuals (including demographic rates such as mortality and growth), (3) population dynamic effects (abundance, population growth), (4) community effects (species richness, diversity, trophic structure), and (5) effects on ecosystem processes (nutrient availability, primary productivity). The genetic effects are rather a special case, although the risk of hybridization with a native congener or other existing biological control agent can exist [31]. The remaining effects form a hierarchy of increasing impact from minimal to massive, detailing for each of the 5 descriptors, an impact or not, at every level from the individual plant to the ecosystem. Successful weed biological control can have indirect beneficial effects such as increased economic productivity, restored community or vegetation structure and ecosystem processes, and improved management effectiveness [32].

The risk assessment for weed biological control agents has seen standards rise over time, with increasing conservatism due to factors such as the Convention on Biodiversity [33]. Risk assessment also varies between jurisdictions. One of the most highly-regarded regimes is that in New Zealand [33], under the Hazardous Substances and New Organisms Act (1996) [34]. The risk assessment for non-target impacts includes a consideration of beneficial and adverse effects. We have limited our consideration to adverse non-target impacts, which should logically take into account the impacts on individual plant taxa, irrespective of how many agents have been involved. We have reviewed the reported non-target impacts on plant species and assessed their magnitude of adverse impact on a five step scale that we have adapted from use with invasive species.

## Methods

### Updating the number of biological control agents released

In the 20^th^ century, 1,120 releases of 365 species of biological control agents were made against 133 weeds in 75 countries [35], predominantly USA, Canada, Australia, South Africa and New Zealand. We reviewed the literature and contacted experts to identify a further 147 agents (Table 1), generating a new total of 512 organisms released for weed biological control, to May 2012.

**Table 1:** Updated list of classical biological control agents released against weeds, since Julien and Griffiths [35].

Country	Insects	Mites	Pathogens	Nematodes	Total	Source
South Africa	32	1	3		36	[74]
Canada	11				11	[75]; R. Bourchier pers. com.
New Zealand	18				18	[34], [49], [76]
Australia	42	3	6		51	R. Winston, pers. com.
European Union	2				2	[77], [78]
USA/Hawai'i	24	1	3	1	29	E. Coombs, pers. com.;[79]
Total	129	5	12	1	147	

### Risk assessment scale

The Environmental Risk Management Authority of New Zealand (1996–2011) (and its successor, the Environmental Protection Authority, 2011-) uses a five step scale for risk assessment of new organisms such as weed biological control agents [36], for which we have proposed modifications to the accompanying text (Table 2), generated from known types of ecological consequences of invasive species [15]. Further, we propose that only items at moderate or above impact are to be considered “significant”, since these definitions are based on plant populations declining, which we believe is a crucial point. Short-term impacts on individual plants with recovery should not receive the same weighting as impacts involving plant population decline, and this point is treated in detail here.

**Table 2:** Proposed scale for retrospectively assessing the magnitude of adverse environmental effects from biological control introductions.

Descriptor	Effects on individuals	Population dynamic effects	Community effects	Effects on ecosystem processes
**Minimal**	Feeding on non-target occasionally recorded, little successful development	-	-	-
**Minor**	Feeding damage	Seasonal feeding on non-target of <50% individuals, plant recovery	-	-
**Moderate**	Impact on fitness	Self sustaining population established on non-target, plant reproduction affected at population level	Minor detrimental habitat modification, or adverse effects on other biocontrols	-
**Major**	Plants killed and reduced reproduction	Impact on plant population readily detectable	Habitat modification detectable, impact on other organisms detectable	Minor effects on ecosystem processes
**Massive**	Plants killed before reproduction	Heavy impact and rapid population decline, species loss	Change in habitat structure of keystone species	Plant succession affected, changes to vegetation cover, loss of keystone species, ecosystem disruption

It is based on the system used in New Zealand under the HSNO Act (1996) for consideration of future risk following new organism introductions, redefined after Parker et al. [15].

### Retrospective application of the risk assessment scale

**Figure 1:**
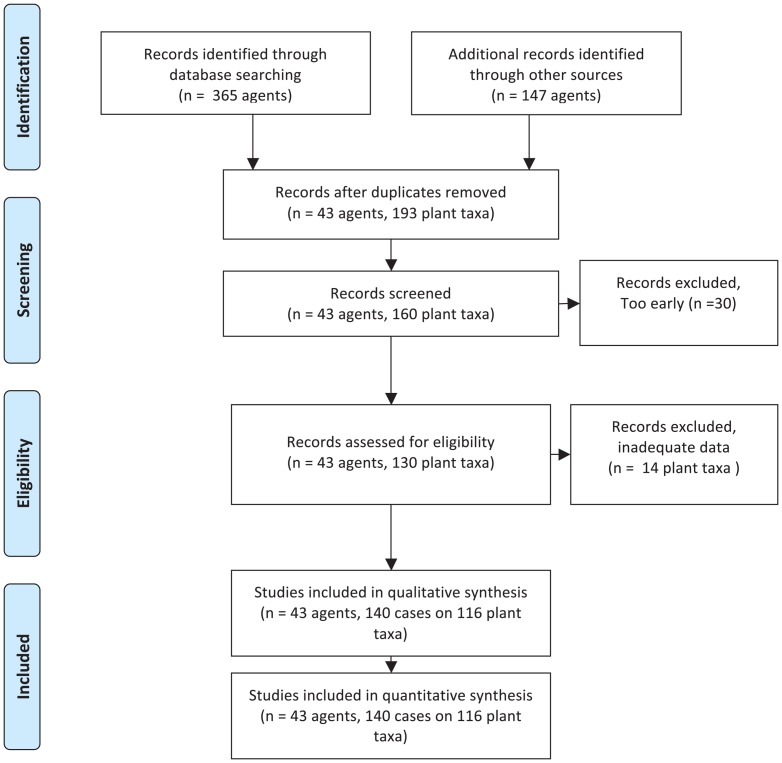
Modified PRISMA flow chart used in the systematic review process [**39**] for non-target impacts from classical biological control of weeds.

A systematic review was conducted of the specialized entomological literature sourced entries for a database of non-target impacts by plant taxon, agent and geographic location, from reviews [21], [22], [27], [37], [38] and primary peer-reviewed reports. In our search for literature to May 2012 we used as descriptors (weeds OR aquatic weeds OR weed control) AND (nontarget organisms OR nontarget effects OR host range OR host preferences OR host specificity OR risk) AND (biological control OR biological control agents) AND (insects). Filtering of results on the agents was recorded in a modified PRISMA chart (Preferred Reporting Items for Systematic Reviews and Meta-Analyses) (Fig. 1) [39], with results recorded by agent and plant taxa in a database (Table S1). Adverse effects were assessed where sufficient information was available (see citations) and each case was assigned a level of adverse impact within a five-step scale from “minimal to massive”, based on Table 2. It was recognised that some minor effects might have been overlooked, but that non-target plant population suppression (moderate, major or massive impact) would probably be observed. Some plants or agents were included more than once as separate cases, with impacts at different magnitudes in different locations, due to parasitism or factors affecting non-target species abundance. Other attributes of each case were recorded, including year of introduction, evidence for the presence of a self-sustaining population, and type of plant (weed, native weed, crop, valued plant). Cases considered of negligible impact were not included (i.e. below minimal), such as the feeding on sunflower (reported in McFadyen [21] requiring cues from pollen [40], and treehoppers present but not feeding on many plants in Brisbane [41]. Self-sustaining populations did not include cases where herbivores established during an initial population explosion from nearby hosts but declined thereafter (e.g. the eriophyid mite *Aculus hyperici* on *Hypericum gramineum* [42]). These cases were considered to be minimal in magnitude, as a result of short-term non-target effects. We noted that some insects considered to be biological control agents were initially self-introduced, but were later deliberately distributed (e.g. *Larinus planus* (F.) Col. Cucurlionidae arrived from Europe by 1971, later released in Canada and the western US). Our analysis of the case of cactus moth *Cactoblastis cactorum* (Bergroth) includes the deliberate introduction to the Caribbean in the presence of native *Opuntia* species which were considered to be weeds in the 1950s, and where impacts have been recorded from its inadvertent and possibly natural spread to Florida [43].

### Assessment of Indirect Effects

We also attempted a separate exercise on adverse indirect effects on ecosystems, but this was more difficult as there was less information, and most cases have only weak evidence, and may not be enduring enough to warrant inclusion (Table 2). It has been recognised for many years that removal of a weed through biological control may lead to either revegetation with native species or simply a change in weed species [44], and this effect was the most common source of indirect effects reported. For indirect effects [32], we have considered cases with an increased abundance of exotic species, only where weed problems were exacerbated. We again used Table 2; by our definition any indirect ecosystem effects start at a magnitude of moderate, with habitat modification.

## Results

### Effects on plants

**Figure 2:**
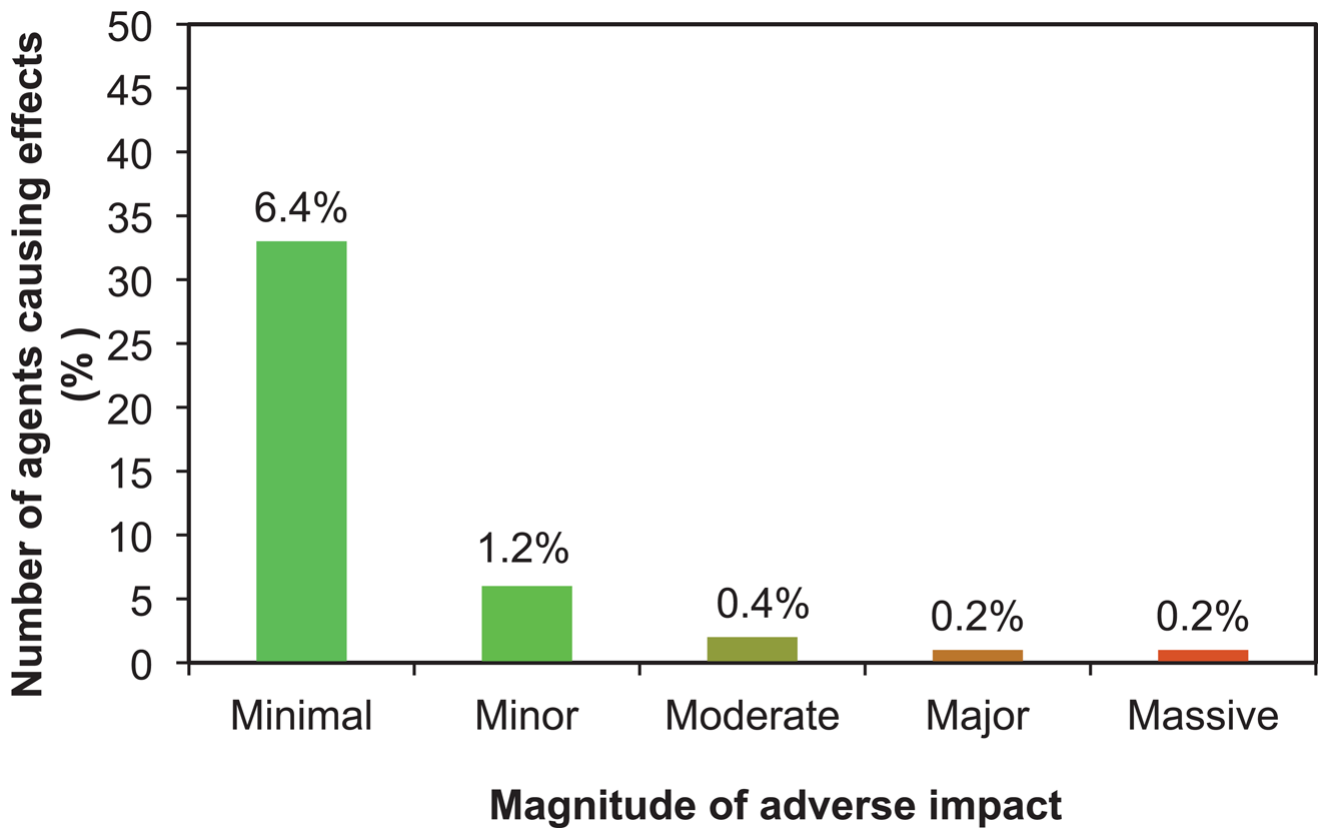
Number of biological control agents causing adverse impacts on non-target plant taxa, by magnitude.

Non-target effects on plants were recorded on a total of 193 cases affecting 152 plant taxa, of which 140 cases on 116 plant taxa were adequate for assessment of magnitude (Fig. 1), from 43 arthropods of 512 used as classical biological control agents of weeds. The details of each case that formed the basis of our assignments can be traced through Table S1, where details are limited here. Case studies to illustrate each magnitude are shown in Table 3, including potentially massive level adverse impacts judged to be underway on one *Opuntia* species in Florida, so far, from *Cactoblastis cactorum*. Major effects were assessed as underway on five other cactus species (*O. cubensis* Britton & Rose, *O. humifusa* (Raf.) Raf., *O. stricta* (Haw.) Haw., *O. triacantha* (Willd.) Sweet, *O. cochenillifera* (L.) Mill.) in either Florida or Nevis and St Kitts [45], as well as on one thistle from thistle seed weevil (Table 3). Effects were assessed as moderate on two other thistles: *Cirsium undulatum* (Nutt.) Spreng. from *Larinus planus* and on *C. altissimum* L. Spreng. from *Trichosirocalus horridus* (Table 2), as well as effects from *C. cactorum* on *Opuntia triacantha* in Nevis and St Kitts [45]. All other impacts that could be assessed were judged as minor or minimal, with no enduring adverse effect on non-target plant populations (Table S1). About 8% of the non-target host plants with reported effects had above minimal-minor adverse impacts, which would be likely to affect plant reproduction (Fig. 2). Percentages in each magnitude are over the total number of non-target plant taxa (N = 140).

**Table 3:** Examples of each magnitude of non-target impact on plants from weed classical biological control agents.

	Target species	Non-target species	Cause & predictability	Potential threat	References
**Minimal**	*Ulex europaeus* L.	*Genista monspessulana* (L.) L.A.S. Johnson	Deliberate release of two populations of *Cydia succedana* (Denis and Schiffermüller), one showed limited development on weeds including this one; Predictable	None, majority of examples	[24], [61]
**Minor**	*Hypericum perforatum* L.	*Hypericum concinnum* Benth	Deliberate release of *Chrysolina quadrigemina* in California causing damage and varying impact on *H. concinnum*, it is still common; Predictable	None, some examples	[80]
**Moderate**	*Carduus nutans* L.	*Cirsium altissimum* L. Spreng	Deliberate release of *Trichosirocalus horridus* causing damage on non-target native thistles; Predictable	Too early to tell; impact is uncertain (moderate impact may be too high); rare.	[81], [82]
**Major**	*Carduus nutans* L.	*Cirsium canescens*, Nutt.& *Carduus*	Deliberate release of *Rhinocyllus conicus* in the U.S. mainland following host range testing proving its safety for crops. Evidence of local population decline of *Cirsium canescens*; Predictable	High likelihood of some attacks on ∼28 species of native thistles; otherwise rare.	[19], [83]
**Massive**	*Opuntia lindheimeri,* Engelm., *O. stricta*, (Haw.) Haw., *O. triacantha* (Willdenow) Sweet	*Opuntia spinosissima* P.Mill.	Accidental release of *Cactoblactis cactorum* in the U.S. mainland following introduction against cacti in the Caribbean; severe feeding impact (threat of extinction without intervention?); Predictable	High likelihood of attacks on ∼87 native cacti species (too early to tell for most); rare.	[27], [45], [46], [70]

A few non-target plants were affected by more than one insect due to multiple introductions against related plants (e.g. *Rubus hawaiiensis* was judged to have had minimal impact from *Priophorus morio* and *Croesia zimmermani*, and minor impact from *Schreckensteinia festaliella* introduced to target other *Rubus*). It is clearly undesirable to have non-target herbivory effects accumulating.

It was considered too early to tell the magnitude of impact for cactus moth attack on most *Opuntia* species in North America, although host range testing appears to place several species at major to massive risk (*Opuntia engelmannii* Salm-Dyck ex Engelm. var. engelmannii, *Opuntia engelmannii* Salm-Dyck ex Engelm. var. linguiformis (Griffiths) Parfitt & Pinkava, *O. ficus-indica* (L.) Mill.,*O. stricta ( = Opuntia dillenii), O. triacantha*), while several other species are likely to have plant resistance (*Consolea rubescens* (Salm-Dyck ex de Candolle), *Cylindropuntia acanthocarpa* (Engelmann and Bigelow) F.M. Knuth, *C. spinosior* (Engelmann), *O. leucotricha* de Candolle and *O. streptacantha* Lem.) [46].

### Effects of insects

**Figure 3:**
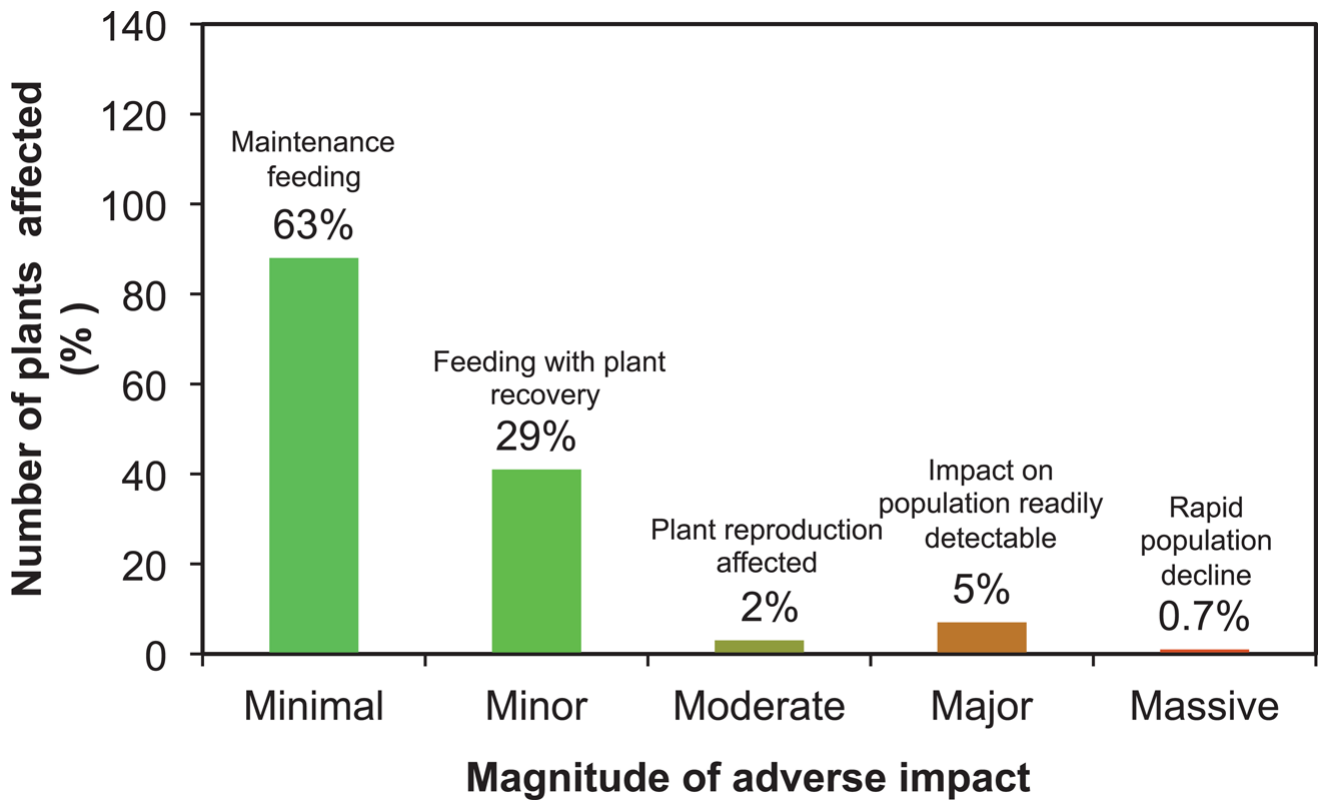
Number of plant taxa with non-target impacts from weed biological control agents, by magnitude.

**Figure 4:**
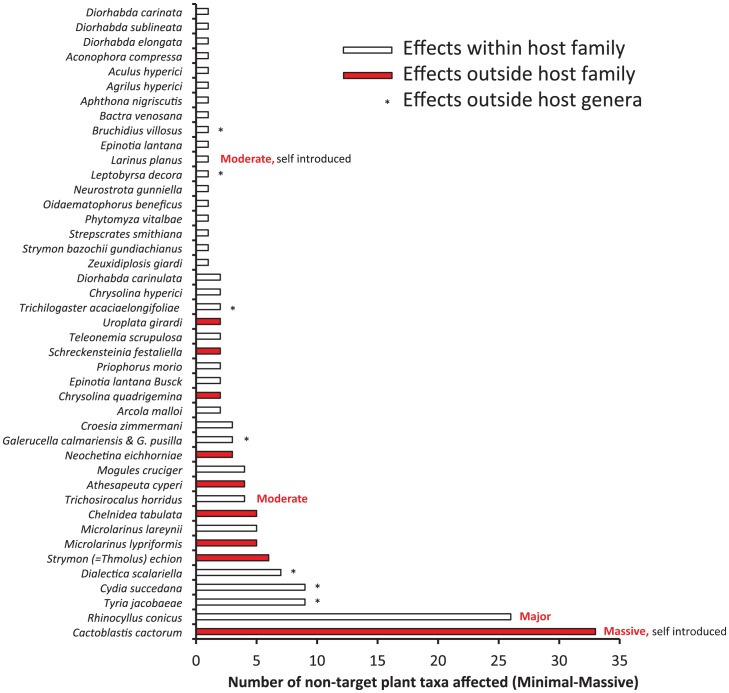
Number and phylogenetic proximity of non-target plant taxa known to be affected by weed biological control agents (minimal-massive).

Of 512 agents released (Table 1 plus [35]), 91.6% of agents have had no known or recorded non-target impact (Fig. 3). Minimal impact (33 agents) or minor impact, with no reduction in plant population (6 agents) occurred with a further 7.6% of agents. Thus non-target plant populations were only adversely affected (moderate-massive range) from 0.8% of agents (n = 4 of 512). The four insect species accounted for all significant adverse effects on non-target plant populations (i.e. moderate-massive, Fig. 4); all were thistles or cacti and within the same genus as the target host plant. Of these, only two were deliberate introductions to the places where they have caused harm (*R. conicus* released in 1969 and thistle rosette weevil *Trichosirocalus horridus* released in 1974), with predictable outcomes that resulted from an earlier era of lower standards of biosafety than prevail today.

A total of 108 of 140 cases of recorded non-target impacts (77%) were in the same plant family as the target weed. About half (54%) were in the same genus as the target (Fig. 4). A few cases of minimal or minor non-target impacts occurred outside the host genus (stars, Fig. 4), or outside the host family (shaded bars, Fig. 4), but impacts in a different plant family are not known to cause plant populations to decline over time (i.e. have not been reported to our knowledge). All of the effects on crops and valued plants (such as ornamentals) were minimal in magnitude.

**Figure 5:**
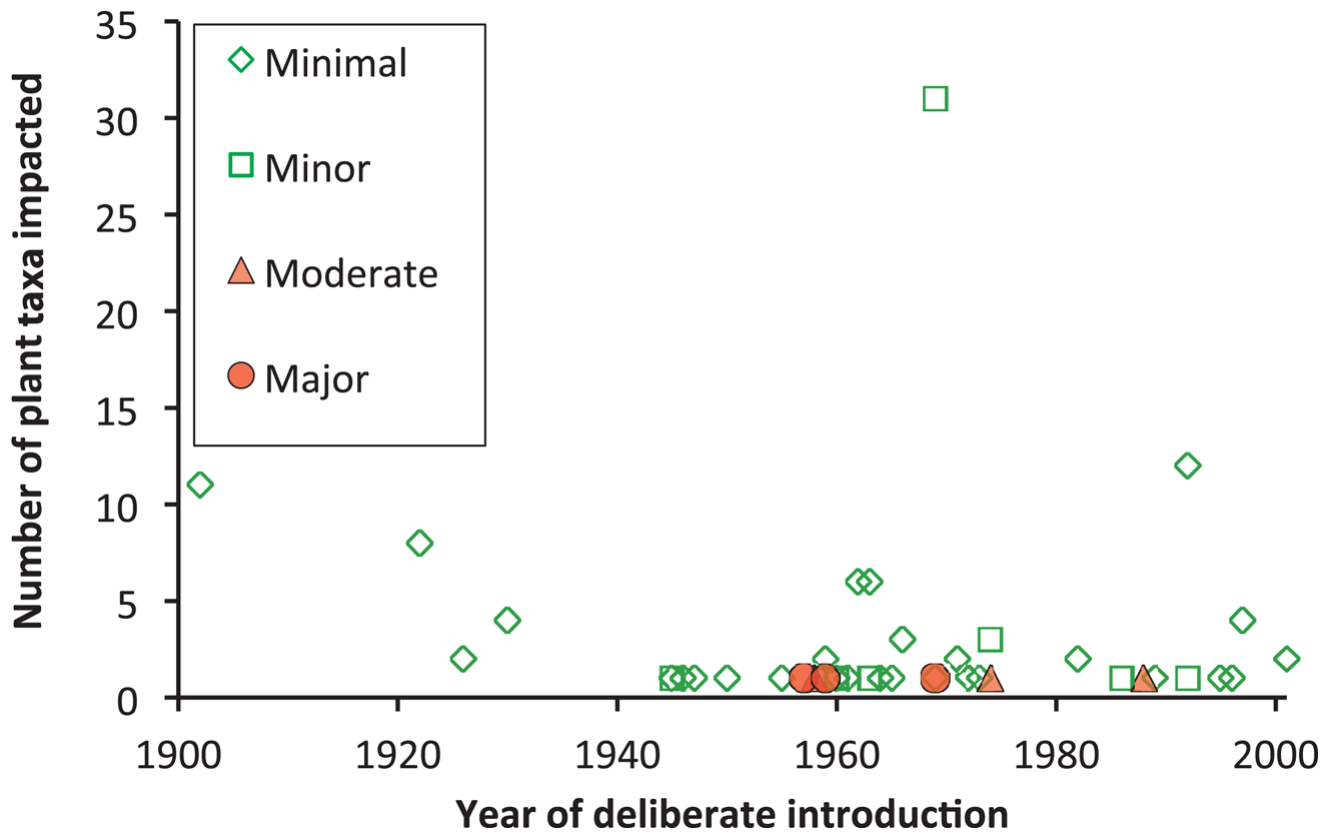
Year of deliberate introduction of arthropods used as weed biological control agents, sorted by magnitude of non-target impact on plant taxa.

For deliberate releases, there were no massive effects determined. Major effects (n = 3 plant taxa) only occurred from deliberate releases in the 1950s–60s in Nevis and St Kitts (*C. cactorum* on *O. stricta* (Haw.) Haw. and *O. cochenillifera* (L.) Mill., and *Rhinocyllus* on *C. canescens* Nutt.) (Fig. 5). Moderate effects occurred from three releases in the period 1958–1988 (n = 3 plant taxa, *C. cactorum* on *O. triacantha* (Willd.) Sweet, *Larinus* on *C. undulatum* (Nutt.) Spreng. and *Trichosirocalus* on *C. altissimum* L. Spreng. Minor effects (n = 39) occurred from releases in the period 1945–1992, while minimal effects occurred from releases in the period 1902–2001 (n = 71). In the case of both *Cactoblastis* and *Rhinocyllus*, a range from major to minimal impacts occurred on different plant taxa. Plant families varied in frequency of reported non-target impacts (Table 4). Table 4 gives a historical view of families with any negative impact from released biocontrol agents but i) does not reflect genetic linkages between plant taxa, and ii) does not rank risk of adverse impact between plant families. The potential obviously exists to further investigate the types of insects and plants showing any adverse effects, including minimal and minor effects, since these cases could be a harbinger of future problems.

**Table 4:** Plant taxa with any non-target impact recorded from classical biological control agents of weeds, sorted by family.

	Number of plant taxa
Asteraceae	47*
Cactaceae	31*
Fabaceae	12
Boraginaceae	7
Cyperaceae	5
Rosaceae	5
Clusiaceae	4
Zygopyhyllaceae	4
Lythraceae	3
Amaranthaceae	2
Hypericaceae	2
Verbenaceae	2
Euphorbiaceae	1
Myricacae	1
Pontederiaceae	1
Ranunculaceae	1

significant impacts (moderate to massive) occurred within plant families.

Four cases of indirect non-target impacts were of moderate to major impact, although two of these we regarded as hypothetical or in progress, in our view awaiting better evidence at the time of writing (Table 5). Some cases involve the agent being effective as originally intended, and moderate effects have resulted from changes in plant cover. We have not included a somewhat similar case [47], as it was unclear whether the removal of ragwort exacerbated the thistles. One case (*Agapeta zoegana*) [48] did not meet our threshold for evidence of a real adverse ecological effect.

**Table 5:** Assessment of potential magnitude of indirect adverse ecological effects from biological control agents (including target weed removal).

Agent	Target	Magnitude	Effect, comment	Reference
*Urophora affinis* and *U. quadri-fasciata*	*Centaurea maculosa*	Moderate	Elevating deer mouse populations, *Peromyscus maniculatus*, hypothetical	[84]
*Diorhabda elongata*	*Tamarix* spp.	Moderate	Loss of saltcedar vegetation^1^ impacting bird nesting, mainly the southwestern willow flycatcher (*Empidonax traillii extimus*), hypothetical	[64]
*Rhinocyllus conicus*	*Carduus nutans*	Moderate	Declining populations of native picture-wing flies when seeds of their native thistle hosts were consumed by *R. conicus*	[85], [86]
*Chrysolina quadrigemina*	*Hypericum perforatum*	Moderate to Major	Aggravating weeds, to *Bromus* spp., *Convovulus arvensis, Centaurea solstitialis, Taeniatherum caput-medusae*, common occurrence	[68]

^1^ The absence of native trees is not the result of the biological control agent.

### Assembling the meta-analysis

**Figure 6:**
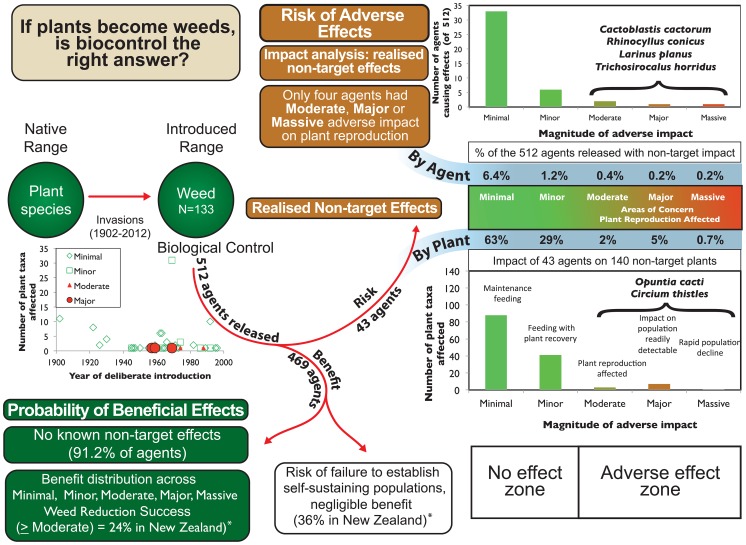
Impact analysis from biological control agents for weeds, with direct adverse impacts observed on non-target plants, and consideration of beneficial effects (*[13]).

No known direct or indirect non-target impacts were found from 91.6% of agents released (Fig. 6). However, some of these agents have failed to establish, representing the risk of failure to achieve benefits. For example, about 36% of agents failed to establish in the history of weed biological control in New Zealand [13]. This risk of failure to get benefits may be declining [49]. Of those that established and had non-target impacts, the majority of these were minimal or minor impacts that had no effect on plant population density. The majority of observed “effects” when considered by plant or by agent are actually in the no effect zone, when impact on non-target plant populations is considered. This leaves a few cases of impacts on plants from four introductions made some decades ago, where reasonably serious adverse non-target effects have been shown within the host genus. Two were deliberate introductions (and predictable) and two were not deliberate. In all of these cases (including those with minimal to minor impact), insect host range mainly spanned genera, although some had lower levels of non-target effects from limited host use outside host families. The benefit side of the equation has not been studied for the full range of agents globally, although two successes with weed biological control were massive in beneficial effect in New Zealand, due to long term ecosystem removal of the target; 24% of New Zealand cases gave some clear benefit against target weeds (moderate-massive) [13].

## Discussion

### Effects on plants

Host range was found to be predictable for biological control agents released over the last century. The risk of host shifting has not been realised with any significant evidence. Population explosions have sometimes occurred at the initial phase of establishment, and occasionally crossed plant family boundaries. Examples of short-term spillovers include local feeding on melons and tomato by the prickly pear biological control agent *Cactoblastis cactorum*, after an initial population explosion and collapse of cacti [50], and *Teleonemia scrupulosa* (Hem.: Tingidae) on sesame when first introduced in East Africa against lantana [51]. For non-target plants receiving minimal or minor impacts, some species were reportedly capable of supporting self-sustaining populations (12/19), but this information was largely missing. This would seem to be a desirable standard for new host records. The effect of spillover onto other plants from initial large insect populations has generally not led to long term impacts on plant population levels, and was rated minimal or minor in magnitude in the vast majority of cases. The exceptions were cases involving four insects (*Cactoblastis*, *Rhinocyllus, Larinus* and *Trichosirocalus*). These cases illustrate the problem, but are not typical.

### Effects of agents

We suggest that weed biological control has a 150 year historical biosafety track record of >99%, as far as is known. We acknowledge that unobserved impacts are possible because of the lack of post-release evaluation studies in most biocontrol programs [52], and we agree that greater efforts are needed to follow up previous introductions for improving assessment of both benefit and risk in future cases. However, the presence of completely unobserved moderate or greater long-term impacts seem unlikely in the majority of cases of releases. The importance of an atypical few (but very frequently cited) cases of major to massive potential impact is partly due to their large number of non-target hosts, as well as a general desire to avoid such non-target impacts. Of the four insects which have caused significant adverse impacts on plant populations, two were not even originally deliberately introduced to the areas most affected. Impacts from the two deliberately introduced insects that have caused impacts (*Rhinocyllus* and *Trichosirocalus*) were foreseeable [27] and would not be permitted today. Not all cases with lower level impacts (minimal-minor) were necessarily within the same family and perhaps predictable, but of the cases with observed non-target feeding across plant families, all were minimal in magnitude. The original host range testing was, in a few cases, inadequate by modern standards, and later testing revealed that the non-target attack was predictable [53]. Retrospective analysis of predictability is not usually an easy task as laboratory host specificity tests are rarely published, but it has to be encouraged for improving risk assessment of potential new invaders [54], [55].

Lawton [56] suggested that Diptera and Lepidoptera make worse biological control agents than Coleoptera and, possibly, Hemiptera. We did not find any evidence that the risk of non-target effects was greater with any particular insect order, as the data are limited to only three beetles and one moth causing significant adverse effects on non-target plant populations, as far as has been recorded.

### The Rule of Tens

The “Rule of Tens” for biological invaders suggested that one tenth of organisms imported established self-sustaining populations in Britain, and of these one tenth became a pest [57]. They considered the case of biological control agents for weeds to be an exception, with greater probability of establishment (61%) and successful pest control (32%) partly due to deliberate release of large numbers. Our results suggest that another interpretation of the rule of tens may be valid for non-target impacts from weed biological control agents, because non-target impacts of any magnitude occurred with about 10% of introduced organisms (8.4%), and ∼1% had an impact on non-target plant population dynamics (i.e. 0.8% became a pest at moderate to massive levels of adversity against the plant), according to our proposed scale.

#### Distribution of impacts

Like weeds, which are sometimes seen as plants growing in the “wrong” place, biological control agents can become invasive. The scaling of magnitude of non-target impacts from biological control agents has not been attempted previously, although there has been much discussion and increasing assessment of the details of an apparently small number of such cases in recent decades [18]–[20]. The visibility and amplitude of the debate has raised the risk of reducing efforts on biological control and achieving fewer net benefits in future. This is a risk because of the increased costs of providing better evidence of biological safety, with a demand for investigation of increasingly subtle effects, such as apparent competition, trophic cascades, and indirect mutualisms [20]. While these are putatively valid mechanisms for non-target impact, the available evidence for their importance as the source of indirect effects from insects introduced for weed biological control comes from increasingly complex experimental manipulations of multiple trophic levels [48], [58]. While such complex interactive effects are of ecological and potentially management interest, the full effects and duration of impact remain unknown. As an example, the gall flies introduced against knapweed caused increased densities of native deer mice [20], which presumably could be beneficial for their predators, but logically this will last only until the knapweed declines or something else changes. Interactions between multiple biocontrol agents or trophic levels can sometimes produce negative management outcomes, although these types of interactions are dynamic [32]. Fowler and Withers [59] could find no indirect effects from weed biocontrol agents in New Zealand mediated via increased populations of natural enemies that exploit the introduced agent. The possibility of indirect competition may exist, but not be realised [60]. Such effects are unlikely to be large unless a “keystone species” is involved, but further analysis might indicate the frequency of this situation [16].

### Predictability and systematic

Paynter et al. [53] identified several cases of inadequate procedures for host range testing which can explain some failures to predict field results. In the vast majority of cases, the laboratory-derived host range is wider than the observed host range in the field [61], and our results support the premise of predictability of insect host range in the field for the types of organisms considered for weed biological control. In some cases, beneficial collateral damage on weeds has occurred, as in the case of *Cydia succedana* (Lep.: Tortricidae), released into New Zealand after host range testing of one provenance. The situation, judged here to have minimal or minor non-target impacts (Table S1), is now understood as release of a mixture of two sources of the insect with different host ranges, highlighting the critical importance of adequate systematic support [24], as well as only releasing insects from sources which have been tested, as is now required in New Zealand.

After being famous for suppressing weedy cacti in Australia, cactus moth has become infamous as it heads to the *Opuntia*-rich regions of the southwestern USA and Mexico [62], [63] and threatens one of the rarest plants in North America (Table 3). However, investigations on Nevis and St Kitts 50 years after the deliberate introduction (which found no extinctions) [45] and host range tests on a range of species [46] suggest that there will be significant differences between species and populations attacked by cactus moth.

### The unpredictability of trophic cascades

Decisions to introduce new organisms can have adverse indirect consequences on weeds which have beneficial effects from supporting birds (Table 5). Many bird species, such as the endangered willow flycatcher, use saltcedar as breeding habitat [64]. Local reduction in saltcedar populations reduced nesting habitat [65], and this led the U.S. to adopt a moratorium on interstate movement of the agent [64]. However, the ecological indirect effects of the insects on saltcedar and bird populations are complex, with both positive effects (beetles used as prey), negative effects (loss of riparian habitat), and no change [65]. We have not found any evidence reported of the effects of defoliation on nest failure. This example illustrates the complexity of indirect ecosystem effects in weed biocontrol where birds are considered as keystone species [66], benefiting to plants with a significant reduction observed in the level of leaf damage and plant mortality [67].

### A weed after a weed

Non-target impacts from successful weed removal can include a shift from exotic species to native vegetation (obviously a desired outcome), or a result in a shift to other exotic weeds. If the new exotic weeds are worse, then biological control has had moderate or higher adverse indirect impact. If the weeds are equivalent, then there has been no obvious gain or loss, just an exchange of species [68]. The limited cases of weed succession listed in Table 5 reflect the scarcity of scientific data when ecological impacts occur at different levels of the trophic cascade. The shifting heterogeneity according to the geographic location described by Campbell and McCaffrey after the removal of Saint John's wort [68] shows that a multi-factorial approach (i.e. climatic, geological, edaphic, etc.) is necessary to understand subtle ecological processes. Observed ecological changes were not predicted before release of biological control agents, at least in a few cases.

### To spread or not to spread?

Controlling the spread of a biological control agent becomes a double-edged sword because the potential benefits are reduced. Because of the magnitude of their impacts, *Cactoblastis*, *Rhinocyllus, Larinus* and *Trichosirocalus* raise wider questions for the feasibility of limiting redistribution of efficient biological control agents from one region to another. These cases posit the question: can we expect to limit the spread of any biological control agents to areas where their targets are weeds, not valued plants? These are cases of biological control where the servant has become a pest, and the search is underway for biocontrol agents of a biocontrol agent [69], the cactus moth. This possibility has been considered for some time [70], but will be complex because of native pyralid moths which could be placed at risk, as well as the risk of loss of weed biological control where it has been deemed desirable, such as Australia.

It is unclear how many of the thistles attacked are actually at risk of declining from the thistle seed weevil, but there is also a further risk of spread to the very rare Pitcher's thistle (*C. pitcheri*) should it disperse or be distributed into this rare plant's protected habitat [19], [71]. Prudent conservation management suggests attempting to limit the further spread of such species which are capable of having significant non-target impacts, but this will limit benefits also. It seems probable that the existing worst four cases above will have extended host range utilization beyond the known (mostly congeneric) level under greater examination and geographic spread, as new similar hosts are encountered. Given scarcity of evidence for wide significant adverse impacts, it seems less likely that the number of significantly impacted hosts of other insects will expand rapidly upon greater scrutiny. New cases of non-target impact on plant populations can be expected to generally follow the observed distribution.

Our approach offers the benefit of providing a standardised framework for observing change in impact over time, since a number of effects are likely to be in flux, for example due to expanding geographic range. The application of the same five step scale used here to characterise benefits from weed biological control at the national level in New Zealand concluded that 24% of agents were successful at weed population suppression (the goal), and two cases were massive in benefit (long-term benefit at ecosystem scale) [13]. This goal needs to be balanced against the risks of non-target effects, which this study has examined and found to be low, if operated based on modern scientific methods. This is despite selected examples that indicate the problem (a tiny proportion of insects introduced for weed biological control are largely predictably adversely affecting two plant families).

## Conclusions

It does appear that nearly all risk of significant non-target usage is borne by native plant species that are closely-related to target weeds, as suggested previously [27]. Non-target effects of any significance to plant populations arise only very rarely after more than a century of classical biological control of weeds. The risk of extinction of non-target cacti and thistles is an undesirable consequence of this human activity, but from a risk management perspective, classical biological control of weeds rates very low indeed compared with the environmental effects of invasive species from globalization, climate change, land use change and other human-induced factors which are rapidly accelerating the risks to rare and endangered species everywhere.

Unforeseen feeding outside the families of target host plants, although a recognised phenomenon in laboratory screening [72], has only proven to cause minimal or minor adverse impacts in the field, which are inconsequential to non-target plant population dynamics. The general lack of host shifts beyond the target plant family by weed biological control agents corroborates the proposition that most insects do not feed across more than one or two plant families [73], although polyphagy exists. Hence the choice of agents with a narrow host range and few or no native congeners to the target should mitigate the largest risks. This may lead weed classical biological control programs e.g. [74]-[79] to prioritise weed species with no direct congeners in the invasive range. Furthermore, choosing weed targets with few relatives anywhere would mitigate the risk of unforeseen movement. It seems likely that a review of the degree of genetic isolation in weed biocontrol targets from valued taxa would help to identify whether this is a valid approach to minimize non-target risks. In addition, consideration of the insect and plant families involved in non-target effects warrants further effort. Ecological cascades also require further investigation.

## Supporting Information

Table S1 **Non-target impacts recorded from biological control agents of weeds on other plants, on a five step scale of magnitude.**
(DOCX)
