# Supplementary material for: What Magnitude Are Observed Non-Target Impacts from Weed Biocontrol?
Source: PLoS One. 2014 Jan 13;9(1):e84847. doi: 10.1371/journal.pone.0084847 (PMC3890286; doi:10.1371/journal.pone.0084847)
Supplement: Table S1 — Non-target impacts recorded from biological control agents of weeds on other plants, on a five step scale of magnitude. (DOCX) [file pone.0084847.s001.docx]

**Table S1. Non-target impacts recorded from biological control agents of weeds on other plants, on a five step scale of magnitude**.

| **Target weed** | **Family** | **Common name** | **Biological Control Agent** | **Family** | **Location released** | **Release date** | **Non-targets attacked** | **Family** | **Impact** | **Source** |
| --- | --- | --- | --- | --- | --- | --- | --- | --- | --- | --- |
| Acacia longifolia (Andrews) Willdenow | Fabaceae | Sydney golden wattle | Trichilogaster acaciaelongifoliae  Frogatt | Pteromalidae | South Africa | 1982 | Acacia melanoxylon Brown | Fabaceae | Minimal | [1] |
| Acacia longifolia (Andrews) Willdenow | Fabaceae | Sydney golden wattle | Trichilogaster acaciaelongifoliae  Frogatt | Pteromalidae | South Africa | 1982 | Paraserianthes lophantha (Willdenow) Nielson | Fabaceae | Minimal | [1,2] |
| Ageratina riparia (Regel) R.M.King & H.Rob. | Asteraceae | mistflower | Oidaematophorus beneficus  Yano and Heppner | Pterophoridae | HI | 1973 | Ageratitta adenophora | Asteraceae | Minimal | [3] |
| Alternanthera philoxeroides (Mart.) Griseb. | Amaranthaceae | Alligator weed | Arcola (=Vogtia) malloi  (Pastrana) | Pyralidae | FL | 1971 | Alternanthera flavescens Kunth | Amaranthaceae | Minimal | [4] |
| Alternanthera philoxeroides (Mart.) Griseb. | Amaranthaceae | Alligator weed | Arcola (=Vogtia) malloi  (Pastrana) | Pyralidae | LA, TX | 1971 | Blutaparon (= Philoxerus) vermiculare L. | Amaranthaceae | Minimal | [4] |
| Carduus nutans L. and C. macrocephalus Desf. | Asteraceae | Thistles | Trichosirocalus horridus  (Panzer) | Curculionidae | NE | 1974 | Cirsium altissimum L. Spreng. | Asteraceae | Moderate | [5-7] |
| Carduus nutans L. and C. macrocephalus Desf. | Asteraceae | Thistles | Trichosirocalus horridus  (Panzer) | Curculionidae | VA | 1974 | Cirsium discolor (Muhl. ex Willd.) Spreng. | Asteraceae | Minor | [4,8] |
| Carduus nutans L. and C. macrocephalus Desf. | Asteraceae | Thistles | Trichosirocalus horridus  (Panzer) | Curculionidae | NE | 1974 | Cirsium flodmanii (Rydb.) Arthur | Asteraceae | Minor | [9] |
| Carduus nutans L. and C. macrocephalus Desf. | Asteraceae | Thistles | Trichosirocalus horridus  (Panzer) | Curculionidae | NE | 1974 | Cirsium undulatum (Nutt.) Spreng. | Asteraceae | Minor | [9] |
| Carduus spp. Cirsium spp. | Asteraceae | Thistles | Rhinocyllus conicus  Frol. | Curculionidae | MT,WY, NE | 1969 | Cirsium canovirens (Rydb.) Petr. | Asteraceae | Minor | [9] |
| Carduus spp. Cirsium spp. | Asteraceae | Thistles | Rhinocyllus conicus  Frol. | Curculionidae | CA | 1969 | Cirsium andersonii (A. Gray) Petr. | Asteraceae | Minor | [4,10] |
| Carduus spp. Cirsium spp. | Asteraceae | Thistles | Rhinocyllus conicus  Frol. | Curculionidae | CA | 1969 | Cirsium brevistylum Cronquist | Asteraceae | Minor | [9,10] |
| Carduus spp. Cirsium spp. | Asteraceae | Thistles | Rhinocyllus conicus  Frol. | Curculionidae | CO | 1969 | Cirsium calcareum (M.E. Jones) Woot. & Standl. | Asteraceae | Minor | [9,10] |
| Carduus spp. Cirsium spp. | Asteraceae | Thistles | Rhinocyllus conicus  Frol. | Curculionidae | CA | 1969 | Cirsium californicum (Nutt.) Jeps. | Asteraceae | Minor | [10] |
| Carduus spp. Cirsium spp. | Asteraceae | Thistles | Rhinocyllus conicus  Frol. | Curculionidae | CA | 1969 | Cirsium callilepis (Greene) Jeps. | Asteraceae | Minor | [9,10] |
| Carduus spp. Cirsium spp. | Asteraceae | Thistles | Rhinocyllus conicus  Frol. | Curculionidae | NE,WY | 1969 | Cirsium canescens Nutt. | Asteraceae | Major | [10,11] |
| Carduus spp. Cirsium spp. | Asteraceae | Thistles | Rhinocyllus conicus  Frol. | Curculionidae | CA | 1969 | Cirsium ciliolatum (L.F. Hend.) J.T. Howell | Asteraceae | Minor | [10] |
| Carduus spp. Cirsium spp. | Asteraceae | Thistles | Rhinocyllus conicus  Frol. | Curculionidae | CA | 1969 | Cirsium cymosum (Greene) J.T. Howell | Asteraceae | Minor | [10] |
| Carduus spp. Cirsium spp. | Asteraceae | Thistles | Rhinocyllus conicus  Frol. | Curculionidae | CA | 1969 | Cirsium douglasii DC. | Asteraceae | Minor | [10] |
| Carduus spp. Cirsium spp. | Asteraceae | Thistles | Rhinocyllus conicus  Frol. | Curculionidae | CO | 1969 | Cirsium eatonii (A. Gray) B.L. Rob. | Asteraceae | Minor | [9,10] |
| Carduus spp. Cirsium spp. | Asteraceae | Thistles | Rhinocyllus conicus  Frol. | Curculionidae | OR | 1969 | Cirsium edule Nutt. | Asteraceae | Minor | [10] |
| Carduus spp. Cirsium spp. | Asteraceae | Thistles | Rhinocyllus conicus  Frol. | Curculionidae | WY | 1969 | Cirsium flodmanii (Rydb.) Arthur | Asteraceae | Minor | [9,10] |
| Carduus spp. Cirsium spp. | Asteraceae | Thistles | Rhinocyllus conicus  Frol. | Curculionidae | CA | 1969 | Cirsium fontinale (Greene) Jeps. | Asteraceae | Minor | [10] |
| Carduus spp. Cirsium spp. | Asteraceae | Thistles | Rhinocyllus conicus  Frol. | Curculionidae | CA | 1969 | Cirsium hydrophilum (Greene) Jeps | Asteraceae | Minor | [10] |
| Carduus spp. Cirsium spp. | Asteraceae | Thistles | Rhinocyllus conicus  Frol. | Curculionidae | MT,WY, NE | 1969 | Cirsium neomexicanum [Gray](http://en.wikipedia.org/wiki/Asa_Gray) | Asteraceae | Minor | [9] |
| Carduus spp. Cirsium spp. | Asteraceae | Thistles | Rhinocyllus conicus  Frol. | Curculionidae | CA | 1969 | Cirsium occidentale (Nutt.) Jeps | Asteraceae | Minor | [9,10] |
| Carduus spp. Cirsium spp. | Asteraceae | Thistles | Rhinocyllus conicus  Frol. | Curculionidae | MT,WY, NE | 1969 | Cirsium occidentale (Nutt.) Jeps. | Asteraceae | Minor | [9] |
| Carduus spp. Cirsium spp. | Asteraceae | Thistles | Rhinocyllus conicus  Frol. | Curculionidae | MT,WY, NE | 1969 | Cirsium ochrocentrum [A.Gray](http://en.wikipedia.org/wiki/Asa_Gray) | Asteraceae | Minor | [9] |
| Carduus spp. Cirsium spp. | Asteraceae | Thistles | Rhinocyllus conicus  Frol. | Curculionidae | CO | 1969 | Cirsium ownbeyi Welsh | Asteraceae | Minor | [4,10] |
| Carduus spp. Cirsium spp. | Asteraceae | Thistles | Rhinocyllus conicus  Frol. | Curculionidae | CA | 1969 | Cirsium pastoris J.T. Howell | Asteraceae | Minor | [4,10] |
| Carduus spp. Cirsium spp. | Asteraceae | Thistles | Rhinocyllus conicus  Frol. | Curculionidae | MT,WY, NE | 1969 | Cirsium peckii L.F. Hend. | Asteraceae | Minor | [9] |
| Carduus spp. Cirsium spp. | Asteraceae | Thistles | Rhinocyllus conicus  Frol. | Curculionidae | MT,WY, NE | 1969 | Cirsium perplexans (Rydb.) Petr. | Asteraceae | Minor | [9] |
| Carduus spp. Cirsium spp. | Asteraceae | Thistles | Rhinocyllus conicus  Frol. | Curculionidae | MT,WY, NE | 1969 | Cirsium proteanum J. T. Howell | Asteraceae | Minor | [9,10] |
| Carduus spp. Cirsium spp. | Asteraceae | Thistles | Rhinocyllus conicus  Frol. | Curculionidae | CA | 1969 | Cirsium quercetorum (A. Gray) Jeps | Asteraceae | Minor | [4,10] |
| Carduus spp. Cirsium spp. | Asteraceae | Thistles | Rhinocyllus conicus  Frol. | Curculionidae | CO | 1969 | Cirsium remotifolium (=C. centaureae) (Hook.) DC | Asteraceae | Minor | [4,10] |
| Carduus spp. Cirsium spp. | Asteraceae | Thistles | Rhinocyllus conicus  Frol. | Curculionidae | WY | 1969 | Cirsium scariosum Nutt. | Asteraceae | Minor | [4,9] |
| Carduus spp. Cirsium spp. | Asteraceae | Thistles | Rhinocyllus conicus  Frol. | Curculionidae | MT,WY, NE | 1969 | Cirsium scopulorum (Greene) Cockerell ex Daniels | Asteraceae | Minor | [9] |
| Carduus spp. Cirsium spp. | Asteraceae | Thistles | Rhinocyllus conicus  Frol. | Curculionidae | MT,WY, NE | 1969 | Cirsium subniveum Rydb. | Asteraceae | Minor | [9] |
| Carduus spp. Cirsium spp. | Asteraceae | Thistles | Rhinocyllus conicus  Frol. | Curculionidae | CA,WY | 1969 | Cirsium tioganum (Congd.) Petrak | Asteraceae | Minor | [4,10] |
| Carduus spp. Cirsium spp. | Asteraceae | Thistles | Rhinocyllus conicus  Frol. | Curculionidae | MT,WY, NE | 1969 | Cirsium undulatum (Nutt.) Spreng. | Asteraceae | Minor | [4,12,13] |
| Cirsium arvense (L.) Scop. | Asteraceae | Thistles | Larinus planus  (F.) | Curculionidae | Canada | 1988 | Cirsium undulatum (Nutt.) Spreng. | Asteraceae | Moderate | [14] |
| Clematis vitalba L. | Ranunculaceae | Old man's beard | Phytomyza vitalbae  Kaltenbach | Agromyzidae | New Zealand | 1996 | Clematis foetida Raoul | Ranunculaceae | Minimal | [15] |
| Cynoglossum officinale L. | [Boraginaceae](http://en.wikipedia.org/wiki/Boraginaceae) | Hounds tooth | Mogules cruciger Herbst | Curculionidae | Canada | 1997 | Cryptantha spiculifera | [Boraginaceae](http://en.wikipedia.org/wiki/Boraginaceae) | Minimal | [16] |
| Cynoglossum officinale L. | [Boraginaceae](http://en.wikipedia.org/wiki/Boraginaceae) | Hounds tooth | Mogules cruciger Herbst | Curculionidae | Canada | 1997 | Hackelia floribunda | [Boraginaceae](http://en.wikipedia.org/wiki/Boraginaceae) | Minimal | [16] |
| Cynoglossum officinale L. | [Boraginaceae](http://en.wikipedia.org/wiki/Boraginaceae) | Hounds tooth | Mogules cruciger Herbst | Curculionidae | Canada | 1997 | Lithospermum ruderale | [Boraginaceae](http://en.wikipedia.org/wiki/Boraginaceae) | Minimal | [16] |
| Cynoglossum officinale L. | [Boraginaceae](http://en.wikipedia.org/wiki/Boraginaceae) | Hounds tooth | Mogules cruciger Herbst | Curculionidae | Canada | 1997 | Lappula squarrosa | [Boraginaceae](http://en.wikipedia.org/wiki/Boraginaceae) | Minimal | [16] |
| Cyperus rotundus L | Cyperaceae | Purple nutsedge | Athesapeuta cyperi  Marshall | Curculionidae | HI | 1922 | Cyperus alternifolius L | Cyperaceae | No field data | [17] |
| Cyperus rotundus L | Cyperaceae | Purple nutsedge | Athesapeuta cyperi  Marshall | Curculionidae | HI | 1922 | Cyperus esculentus L | Cyperaceae | Minimal | [17] |
| Cyperus rotundus L | Cyperaceae | Purple nutsedge | Athesapeuta cyperi  Marshall | Curculionidae | HI | 1922 | Cyperus papyrus L | Cyperaceae | No field data | [17] |
| Cyperus rotundus L | Cyperaceae | Purple nutsedge | Bactra venosana  (Zeller) | Tortricidae | HI | 1922 | Cyperus esculentus L | Cyperaceae | Minimal | [17] |
| Cyperus rotundus L. | Cyperaceae | Purple nutsedge | Athesapeuta cyperi  Marshall | Curculionidae | HI | 1922 | Cyperus polystachyos Rottb. | Cyperaceae | Minimal | [4,17] |
| Cytisus scoparius Fabricius Link | Fabaceae | Scotch broom | Bruchidius villosus  Fabricius | Bruchidae | New Zealand | 1986 | Chamaecytisus palmensis (Christ) Bisby (=proliferus subspp. palmensis ) | Fabaceae | Minor | [15,18,19] |
| Echium plantagineum L. | Boraginaceae | Patterson’s curse | Dialectica scalariella  (Zeller) | Pterophoridae | NA, Accidental from Australia |  | Borago sp. | Boraginaceae | Minimal | [1] |
| Echium plantagineum L. | Boraginaceae | Patterson’s curse | Dialectica scalariella  (Zeller) | Pterophoridae | NA, Accidental from Australia |  | Cynoglossum sp. | Boraginaceae | Minimal | [1] |
| Echium plantagineum L. | Boraginaceae | Patterson’s curse | Dialectica scalariella  (Zeller) | Pterophoridae | NA, Accidental from Australia |  | Echium spp. | Boraginaceae | Minimal | [1] |
| Echium plantagineum L. | Boraginaceae | Patterson’s curse | Dialectica scalariella  (Zeller) | Pterophoridae | NA, Accidental from Australia |  | Echium vulgare L. | Boraginaceae | Minimal | [1,20] |
| Echium plantagineum L. | Boraginaceae | Patterson’s curse | Dialectica scalariella  (Zeller) | Pterophoridae | NA, Accidental from Australia |  | Echium wildpretIi | Boraginaceae | Minimal | [1] |
| Echium plantagineum L. | Boraginaceae | Patterson’s curse | Dialectica scalariella  (Zeller) | Pterophoridae | NA, Accidental from Australia |  | Myosotis spp. | Boraginaceae | Minimal | [1] |
| Echium plantagineum L. | Boraginaceae | Patterson’s curse | Dialectica scalariella  (Zeller) | Pterophoridae | NA, Accidental from Australia |  | Symphytum sp. | Boraginaceae | Minimal | [1] |
| Eichhornia crassipes (Martius) | Pontederiaceae | Water hyacinth | Neochetina eichhorniae  Warner | Curculionidae | USA | 1972 | Canna sp. | Cannaceae | Minimal | [1,21] |
| Eichhornia crassipes (Martius) | Pontederiaceae | Water hyacinth | Neochetina eichhorniae  Warner | Curculionidae | USA | 1972 | Portaderia cordata L. | Pontederiaceae | No field data | [1] |
| Eichhornia crassipes (Martius) | Pontederiaceae | Water hyacinth | Neochetina eichhorniae  Warner | Curculionidae | USA | 1972 | Unspecified grass | Poaceae | No field data | [1] |
| Euphorbia esula L. | Euphorbiaceae | leafy spurge | Aphthona nigriscutis  Foudras | Chrysomelidae | WY | 1992 | Euphorbia robusta Engelm. | Euphorbiaceae | Minimal | [22] |
| Hypericum perforatum L. | Clusiaceae | St John’s Wort | Aculus hyperici Liro | Eriophyidae | Australia | 1991 | Hypericum gramineum Forst. | Hypericaceae | Minor | [23] |
| Hypericum perforatum L. | Clusiaceae | St Johns Wort | Agrilus hyperici  (Creutzer) | Buprestidae | CA | 1946 | Hypericum concinnum Benth. | Clusiaceae | Minimal | [24] |
| Hypericum perforatum L. | Clusiaceae | St John’s Wort | Chrysolina hyperici  (Forster) | Chrysomelidae | New Zealand | 1947 | Hypericum androsaemum Linneaus | Hypericaceae | Minimal | [1,20] |
| Hypericum perforatum L. | Clusiaceae | St John’s Wort | Chrysolina hyperici  (Forster) | Chrysomelidae | HI | 1965 | Hypericum degeneri Fosb. | Hypericaceae | Minimal | [25] |
| Hypericum perforatum L. | Clusiaceae | St Johns Wort | Chrysolina quadrigemina  (Suffrian) | Chrysomelidae | CA | 1945 | Hypericum calycinum | Clusiaceae | Minimal | [26] |
| Hypericum perforatum L. | Clusiaceae | St Johns Wort | Chrysolina quadrigemina  (Suffrian) | Chrysomelidae | CA | 1945 | Hypericum concinnum Benth. | Clusiaceae | Minor | [24,26] |
| Hypericum perforatum L. | Clusiaceae | St Johns Wort | Zeuxidiplosis giardi  (Kieffer) | Chrysomelidae | USA | 1950 | Hypericum concinnum Benth. | Clusiaceae | Minimal | [24,26] |
| Lantana camara L. | Verbenaceae | Lantana | Aconophora compressa  Walker | Membracidae | Australia | 1995 | Citharexylum spinosum L. | Verbenaceae | Minimal | [27,28] |
| Lantana camara L. | Verbenaceae | Lantana | Epinotia lantana  Busck. | Tortricidae | HI | 1902 | Litchi chinensis Sonn. | Sapindaceae | Minimal | [25] |
| Lantana camara L. | Verbenaceae | Lantana | Epinotia lantana  Busck. | Tortricidae | HI | 1902 | Stenoiobium starts (L.) Seem. | Bignoniaceae | Minimal | [25] |
| Lantana camara L. | Verbenaceae | Lantana | Epinotia lantana  Busck. | Tortricidae | HI | 1902 | Tabfbuia chrysantha (Jacq.) Nichols | Bignoniaceae | Minimal | [25] |
| Lantana camara L. | Verbenaceae | Lantana | Leptobyrsa decora  Drake | Tingidae | HI | 1969 | Duranta repens L. | Verbenaceae | Minimal | [25] |
| Lantana camara L. | Verbenaceae | Lantana | Strymon (=Thmolus) echion  L. | Lycaenidae | HI | 1902 | Capsicum annuum L. | Solanaceae | Minimal | [25] |
| Lantana camara L. | Verbenaceae | Lantana | Strymon (=Thmolus) echion  L. | Lycaenidae | HI | 1902 | Cordia sebestena L. | Boraginaceae | Minimal | [25] |
| Lantana camara L. | Verbenaceae | Lantana | Strymon (=Thmolus) echion  L. | Lycaenidae | HI | 1902 | Datura candida (Pers.) | Solanaceae | Minimal | [25] |
| Lantana camara L. | Verbenaceae | Lantana | Strymon (=Thmolus) echion  L. | Lycaenidae | HI | 1902 | Ocimum basilcum L. | Labiaceae | Minimal | [25] |
| Lantana camara L. | Verbenaceae | Lantana | Strymon (=Thmolus) echion  L. | Lycaenidae | HI | 1902 | Solanum nigrum L. | Solanaceae | Minimal | [25] |
| Lantana camara L. | Verbenaceae | Lantana | Strymon (=Thmolus) echion  L. | Lycaenidae | HI | 1902 | Solanum tuberosum L. | Solanaceae | Minimal | [25] |
| Lantana camara L. | Verbenaceae | Lantana | Strymon bazochii gundiachianus  Bates | Lycaenidae | HI | 1902 | Hyptis pectinata L. | Labiaceae | Minimal | [25] |
| Lantana camara L. | Verbenaceae | Lantana | Teleonemia scrupulosa  Stål | Tingidae | HI | 1902 | Myoporum sandwicense (A. DC.) A. Gray | Scrophulariaceae | Minimal | [29,30] |
| Lantana camara L. | Verbenaceae | Lantana | Teleonemia scrupulosa  Stål | Tingidae | East Africa | 1962 | Sesamum indicum L. | Pedaliaceae | Minimal | [31,32] |
| Lantana camara L. | Verbenaceae | Lantana | Uroplata girardi  Pic. | Chrysomelidae | HI | 1961 | Ocimum basilicum L. | Labiaceae | Minimal | [3] |
| Lantana camara L. | Verbenaceae | Lantana | Uroplata girardi  Pic. | Chrysomelidae | Australia | 1966 | Ocimum basilicum L. | Labiaceae | Minimal | [33] |
| Lythrum salicaria L. | Lythraceae | Purple loosestrife | Galerucella calmariensis (L.) / G. pusilla  (Duft.) | Chrysomelidae | USA | 1992 | Decodon verticillatus (L.) Elliott. | Lythraceae | Minimal | [34,35] |
| Lythrum salicaria L. | Lythraceae | Purple loosestrife | Galerucella calmariensis (L.) / G. pusilla  (Duft.) | Chrysomelidae | USA | 1992 | Lythrum alatum Pursh | Lythraceae | No field data | [34] |
| Lythrum salicaria L. | Lythraceae | Purple loosestrife | Galerucella calmariensis (L.) / G. pusilla  L./Duftschmid | Chrysomelidae | USA | 1992 | Lythrum indica L. | Lythraceae | Minimal | [36] |
| Lythrum salicaria L. | Lythraceae | Purple loosestrife | Galerucella calmariensis (L.) / G. pusilla  (Duft.) | Chrysomelidae | USA | 1992 | Potentilla anserine L. | Rosaceae | Minimal | [35,37] |
| Lythrum salicaria L. | Lythraceae | Purple loosestrife | Galerucella calmariensis (L.) / G. pusilla  (Duft.) | Chrysomelidae | USA | 1992 | Rosa multiflora Thumb | Rosaceae | Minimal | [35,37] |
| Mimosa pigra L. | Mimosaceae | Giant sensitive tree | Neurostrota gunniella  [Busck.](http://en.wikipedia.org/wiki/August_Busck) | Gracillariidae | Australia | 1989 | Neptunia major (Benth.) Windler | Fabaceae | Minimal | [38-40] |
| Myrica faya Aiion | Myricaceae | Fire tree | Strepsicrates smithiana  (Walsingham) | Olethreutidae | HI | 1955 | Myrica cerifera L. | Myricaceae | Minimal | [25] |
| Opuntia lindheimeri,Opuntia stricta,Opuntia triacantha | Cactaceae | Prickly pear | Cactoblastis cactorum  (Bergroth) | Pyralidae | NA, accidental from Carribean |  | Consolea rubescens (Salm-  Dyck ex de Candolle) | Cactaceae | Too early, likely minimal | [41-43] |
| Opuntia lindheimeri,Opuntia stricta,Opuntia triacantha | Cactaceae | Prickly pear | Cactoblastis cactorum  (Bergroth) | Pyralidae | NA |  | \| [Cylindropuntia acanthocarpa (Engelm. & J.M. Bigelow) F.M. Knuth](http://plants.usda.gov/java/profile?symbol=CYAC8) \| \| --- \| | Cactaceae | Too early, likely minimal | [43] |
| Opuntia lindheimeri,Opuntia stricta,Opuntia triacantha | Cactaceae | Prickly pear | Cactoblastis cactorum  (Bergroth) | Pyralidae | NA |  | [Cylindropuntia spinosior (Engelm.) F.M. Knuth](http://plants.usda.gov/java/profile?symbol=CYSP8) | Cactaceae | Too early, likely minimal | [43] |
| Opuntia lindheimeri,Opuntia stricta,Opuntia triacantha | Cactaceae | Prickly pear | Cactoblastis cactorum  (Bergroth) | Pyralidae | NA |  | Nopalea cochenillifera (L.) Salm-Dyck | Cactaceae | Too early | [43] |
| Opuntia lindheimeri,Opuntia stricta,Opuntia triacantha | Cactaceae | Prickly pear | Cactoblastis cactorum  (Bergroth) | Pyralidae | NA |  | Opuntia compressa Small | Cactaceae | Too early | [44] |
| Opuntia lindheimeri,Opuntia stricta,Opuntia triacantha | Cactaceae | Prickly pear | Cactoblastis cactorum  (Bergroth) | Pyralidae | NA |  | Opuntia cubensis Britton & Rose | Cactaceae | Major | [4,45] |
| Opuntia lindheimeri,Opuntia stricta,Opuntia triacantha | Cactaceae | Prickly pear | Cactoblastis cactorum  (Bergroth) | Pyralidae | NA |  | Opuntia dillenii (Ker Gawl.) Haw. | Cactaceae | Too early | [43] |
| Opuntia lindheimeri,Opuntia stricta,Opuntia triacantha | Cactaceae | Prickly pear | Cactoblastis cactorum  (Bergroth) | Pyralidae | NA |  | Opuntia engelmannii Salm-Dyck ex Engelm. var. engelmannii | Cactaceae | Too early, likely Massive | [43] |
| Opuntia lindheimeri,Opuntia stricta,Opuntia triacantha | Cactaceae | Prickly pear | Cactoblastis cactorum  (Bergroth) | Pyralidae | NA |  | Opuntia engelmannii Salm-Dyck ex Engelm. var. lindheimeri (Engelm.) Parfitt & Pinkava | Cactaceae | Too early | [43] |
| Opuntia lindheimeri,Opuntia stricta,Opuntia triacantha | Cactaceae | Prickly pear | Cactoblastis cactorum  (Bergroth) | Pyralidae | NA |  | Opuntia engelmannii Salm-Dyck ex Engelm. var. linguiformis (Griffiths) Parfitt & Pinkava | Cactaceae | Too early, likely Massive | [43] |
| Opuntia lindheimeri,Opuntia stricta,Opuntia triacantha | Cactaceae | Sweet prickly pear | Cactoblastis cactorum  (Bergroth) | Pyralidae | NA |  | Opuntia ficus-indica (L.) Mill. | Cactaceae | Too early, likely Massive | [43,44,46] |
| Opuntia lindheimeri,Opuntia stricta,Opuntia triacantha | Cactaceae | Prickly pear | Cactoblastis cactorum  (Bergroth) | Pyralidae | NA |  | Opuntia humfusa (Raf.) Raf. | Cactaceae | Major | [4,45] |
| Opuntia lindheimeri,Opuntia stricta,Opuntia triacantha | Cactaceae | Prickly pear | Cactoblastis cactorum  (Bergroth) | Pyralidae | NA |  | Opuntia leucotricha DC. | Cactaceae | Too early, likely minimal | [43] |
| Opuntia lindheimeri,Opuntia stricta,Opuntia triacantha | Cactaceae | Prickly pear | Cactoblastis cactorum  (Bergroth) | Pyralidae | NA |  | Opuntia macrocentra Engelm. | Cactaceae | Too early | [43] |
| Opuntia lindheimeri,Opuntia stricta,Opuntia triacantha | Cactaceae | Prickly pear | Cactoblastis cactorum  (Bergroth) | Pyralidae | NA |  | Opuntia megacantha Salm-Dyck | Cactaceae | Too early | [44] |
| Opuntia lindheimeri,Opuntia stricta,Opuntia triacantha | Cactaceae | Prickly pear | Cactoblastis cactorum  (Bergroth) | Pyralidae | NA |  | Opuntia microdasys (Lehm.) N.E. Pfeiffer | Cactaceae | Too early | [43] |
| Opuntia lindheimeri,Opuntia stricta,Opuntia triacantha | Cactaceae | Prickly pear | Cactoblastis cactorum  (Bergroth) | Pyralidae | NA |  | Opuntia santa-rita (Griffiths & Hare) Rose | Cactaceae | Too early | [43] |
| Opuntia lindheimeri,Opuntia stricta,Opuntia triacantha | Cactaceae | Prickly pear | Cactoblastis cactorum  (Bergroth) | Pyralidae | NA |  | Opuntia spinosissima Mill. | Cactaceae | Massive | [4,45] |
| Opuntia lindheimeri,Opuntia stricta,Opuntia triacantha | Cactaceae | Prickly pear | Cactoblastis cactorum  (Bergroth) | Pyralidae | NA |  | Opuntia spinulifera Salm-Dyck | Cactaceae | Too early | [44] |
| Opuntia lindheimeri,Opuntia stricta,Opuntia triacantha | Cactaceae | Prickly pear | Cactoblastis cactorum  (Bergroth) | Pyralidae | NA |  | Opuntia streptacantha Lem. | Cactaceae | Too early, likely minimal | [43] |
| Opuntia lindheimeri,Opuntia stricta,Opuntia triacantha | Cactaceae | Prickly pear | Cactoblastis cactorum  (Bergroth) | Pyralidae | NA |  | Opuntia stricta (= Opuntia dillenii) (Ker Gawl.) Haw. | Cactaceae | Too early, likely Massive | [44] |
| Opuntia lindheimeri,Opuntia stricta,Opuntia triacantha | Cactaceae | Prickly pear | Cactoblastis cactorum  (Bergroth) | Pyralidae | NA |  | Opuntia stricta (Haw.) Haw. | Cactaceae | Major | [4,45] |
| Opuntia lindheimeri,Opuntia stricta,Opuntia triacantha | Cactaceae | Prickly pear | Cactoblastis cactorum  (Bergroth) | Pyralidae | NA |  | Opuntia tomentosa Salm-Dyck | Cactaceae | Too early | [44] |
| Opuntia lindheimeri,Opuntia stricta,Opuntia triacantha | Cactaceae | Prickly pear | Cactoblastis cactorum  (Bergroth) | Pyralidae | NA |  | Opuntia triacantha (Willd.) Sweet | Cactaceae | Major | [4,45] |
| Opuntia lindheimeri,Opuntia stricta,Opuntia triacantha | Cactaceae | Prickly pear | Cactoblastis cactorum  (Bergroth) | Pyralidae | NA |  | Opuntia triacantha (Willd.) Sweet | Cactaceae | Too early, likely Massive | [44] |
| Opuntia lindheimeri,Opuntia stricta,Opuntia triacantha | Cactaceae | Prickly pear | Cactoblastis cactorum  (Bergroth) | Pyralidae | NA |  | Opuntia tuna (L.) Mill. | Cactaceae | Too early | [44] |
| Opuntia lindheimeri,Opuntia stricta,Opuntia triacantha | Cactaceae | Prickly pear | Cactoblastis cactorum  (Bergroth) | Pyralidae | NA |  | Opuntia vulgaris auct. non Mill. | Cactaceae | Too early | [44] |
| Opuntia spp. | Cactaceae | Prickly pear | Cactoblastis cactorum  (Bergroth) | Pyralidae | Australia | 1926 | Cucumis melo L. | Cucurbitaceae | Minimal | [1,21] |
| Opuntia spp. | Cactaceae | Prickly pear | Cactoblastis cactorum  (Bergroth) | Pyralidae | Australia | 1926 | Solanum lycopersicum L. | Solanaceae | Minimal | [1] |
| Opuntia spp. | Cactaceae | Prickly pear | Chelnidea tabulata  (Burmeister | Hemiptera | Australia | 1922 | Cucumis melo L. | Cucurbitaceae | Minimal | [1] |
| Opuntia spp. | Cactaceae | Prickly pear | Chelnidea tabulata  (Burmeister | Hemiptera | Australia | 1922 | Phœnix dactylifera, L. | Arecaceae | Minimal | [1] |
| Opuntia spp. | Cactaceae | Prickly pear | Chelnidea tabulata  (Burmeister | Hemiptera | Australia | 1922 | Prunus persica (L.) Batsch | Rosaceae | Minimal | [1] |
| Opuntia spp. | Cactaceae | Prickly pear | Chelnidea tabulata  (Burmeister | Hemiptera | Australia | 1922 | Prunus persica nucipersica | Rosaceae | Minimal | [1,21] |
| Opuntia spp. | Cactaceae | Prickly pear | Chelnidea tabulata  (Burmeister | Hemiptera | Australia | 1922 | Vitis vinifera L. | Vitaceae | Minimal | [1] |
| Opuntia triacantha (Willd.) Sweet | Cactaceae | Prickly pear | Cactoblastis cactorum  (Bergroth) | Pyralidae | Nevis and ST Kitts | 1957 | Opuntia cochenillifera (L.) Mill. | Cactaceae | Major | [41] |
| Opuntia triacantha (Willd.) Sweet | Cactaceae | Prickly pear | Cactoblastis cactorum  (Bergroth) | Pyralidae | Nevis and ST Kitts | 1959 | Opuntia stricta (Haw.) Haw. | Cactaceae | Major | [41] |
| Opuntia triacantha (Willd.) Sweet | Cactaceae | Prickly pear | Cactoblastis cactorum  (Bergroth) | Pyralidae | Nevis and ST Kitts | 1958 | Opuntia triacantha (Willd.) Sweet | Cactaceae | Moderate | [41] |
| Rubus argutus Link. | Rosaceae | Blackberry | Croesia zimmermani  Clarke | Tortricidae | HI | 1964 | Rubus hawaiiensis A. Gray | Rosaceae | Minor | [47] |
| Rubus argutus Link. | Rosaceae | Blackberry | Croesia zimmermani  Clarke | Tortricidae | HI | 1964 | Rubus macraei A. Gray | Rosaceae | Minimal | [4] |
| Rubus argutus Link. | Rosaceae | Blackberry | Priophorus morio  (Lepeletier) | Tenthredindae | HI | 1966 | Rubus hawaiensis A. Gray | Rosaceae | Minimal | [4] |
| Rubus argutus Link. | Rosaceae | Blackberry | Priophorus morio  (Lepeletier) | Tenthredindae | HI | 1966 | Rubus macraei A. Gray | Rosaceae | Minimal | [4] |
| Rubus argutus Link. | Rosaceae | Blackberry | Schreckensteinia festaliella  (Hübner) | Heliodinidae | HI | 1963 | Rubus macraei A. Gray | Rosaceae | Minor | [4] |
| Rubus argutus Link. | Rosaceae | Blackberry | Schreckensteinia festaliella  (Hübner) | Heliodinidae | HI | 1963 | Rubus macraei A. Gray | Rosaceae | Minor | [4] |
| Senecio jacobaea L. | Asteraceae | Ragwort | Tyria jacobaeae  (Linnaeus) | Arctiidae | New Zealand | 1930 | Cineraria sp. | Asteraceae | Minimal | [1] |
| Senecio jacobaea L. | Asteraceae | Ragwort | Tyria jacobaeae  (Linnaeus) | Arctiidae | New Zealand | 1930 | Senecio biserratus Belcher | Asteraceae | Minimal | [48] |
| Senecio jacobaea L. | Asteraceae | Ragwort | Tyria jacobaeae  (Linnaeus) | Arctiidae | OR | 1960 | Senecio integerrimus Nutt. | Asteraceae | Minimal | [49] |
| Senecio jacobaea L. | Asteraceae | Ragwort | Tyria jacobaeae  (Linnaeus) | Arctiidae | Canada | 1959 | Senecio integerrimus Nutt. | Asteraceae | Minimal | [49] |
| Senecio jacobaea L. | Asteraceae | Ragwort | Tyria jacobaeae  (Linnaeus) | Arctiidae | New Zealand | 1930 | Senecio minimus Poir | Asteraceae | Minimal | [1,15] |
| Senecio jacobaea L. | Asteraceae | Ragwort | Tyria jacobaeae  (Linnaeus) | Arctiidae | OR | 1960 | Senecio pseudaureus Rydb. | Asteraceae | No field data | [49] |
| Senecio jacobaea L. | Asteraceae | Ragwort | Tyria jacobaeae  (Linnaeus) | Arctiidae | OR | 1960 | Senecio triangularis Hook. | Asteraceae | Minor | [47,49,50] |
| Senecio jacobaea L. | Asteraceae | Ragwort | Tyria jacobaeae  (Linnaeus) | Arctiidae | Canada | 1959 | Senecio triangularis Hook. | Asteraceae | Minimal | [49] |
| Senecio jacobaea L. | Asteraceae | Ragwort | Tyria jacobaeae  (Linnaeus) | Arctiidae | New Zealand | 1930 | Sonchus oleraceus L. | Asteraceae | Minimal | [1,51] |
| Tamarix | [Tamaricaceae](http://en.wikipedia.org/wiki/Tamaricaceae) | Salt cedar | Diorhabda carinata (Faldermann) | Chrysomelidae | NV, UT, CO,WY, TX, CA | 2001 | Tamarix aphylla | [Tamaricaceae](http://en.wikipedia.org/wiki/Tamaricaceae) | Minimal | [52] |
| Tamarix | [Tamaricaceae](http://en.wikipedia.org/wiki/Tamaricaceae) | Salt cedar | Diorhabda carinulata (Desbrochers) | Chrysomelidae | NV, UT, CO,WY, TX, CA | 2001 | Frankenia | Frakeniaceae | Minimal | [52] |
| Tamarix | [Tamaricaceae](http://en.wikipedia.org/wiki/Tamaricaceae) | Salt cedar | Diorhabda carinulata (Desbrochers) | Chrysomelidae | NV, UT, CO,WY, TX, CA | 2001 | Tamarix aphylla | [Tamaricaceae](http://en.wikipedia.org/wiki/Tamaricaceae) | Minimal | [52] |
| Tamarix | [Tamaricaceae](http://en.wikipedia.org/wiki/Tamaricaceae) | Salt cedar | Diorhabda elongate (Brullé) | Chrysomelidae | TX | 2001 | Tamarix aphylla | [Tamaricaceae](http://en.wikipedia.org/wiki/Tamaricaceae) | Minimal | [52,53] |
| Tamarix | [Tamaricaceae](http://en.wikipedia.org/wiki/Tamaricaceae) | Salt cedar | Diorhabda sublineata (lucas) | Chrysomelidae | TX | 2001 | Tamarix aphylla | [Tamaricaceae](http://en.wikipedia.org/wiki/Tamaricaceae) | Minimal | [52] |
| Tribulus terrestris L. | Zygophyllaccae | Puncturevine | Microlarinus lareynii  (Jacquelin du Val) | Curculionidae | HI | 1962 | Amaranthus spimosus L. | Amaranthaceae | Minimal | [25] |
| Tribulus terrestris L. | Zygophyllaccae | Puncturevine | Microlarinus lareynii  (Jacquelin du Val) | Curculionidae | HI | 1962 | Chenopodium album L. | Chenopodiaceae | Minimal | [25] |
| Tribulus terrestris L. | Zygopyhyllaceae | Puncturevine | Microlarinus lareynii  (Jacquelin du Val) | Curculionidae | AZ | 1962 | Kallstroemia californica (S. Watson) Vail | Zygopyhyllaceae | Minimal | [4] |
| Tribulus terrestris L. | Zygopyhyllaceae | Puncturevine | Microlarinus lareynii  (Jacquelin du Val) | Curculionidae | AZ | 1962 | Kallstroemia grandiflora Torr. ex A. Gray | Zygopyhyllaceae | Minimal | [4] |
| Tribulus terrestris L. | Zygophyllaccae | Puncturevine | Microlarinus lareynii  (Jacquelin du Val) | Curculionidae | HI | 1962 | Malva parviflora L. | Malvaceae | Minimal | [25] |
| Tribulus terrestris L. | Zygophyllaccae | Puncturevine | Microlarinus lypriformis  (Wollaston) | Curculionidae | HI | 1963 | Amaranthus spimosus L. | Amaranthaceae | Minimal | [25] |
| Tribulus terrestris L. | Zygophyllaccae | Puncturevine | Microlarinus lypriformis  (Wollaston) | Curculionidae | HI | 1963 | Chenopodium album L. | Chenopodiaceae | Minimal | [25] |
| Tribulus terrestris L. | Zygopyhyllaceae | Puncturevine | Microlarinus lypriformis  (Wollaston) | Curculionidae | AZ | 1963 | Kallstroemia californica (S. Watson) Vail | Zygopyhyllaceae | Minimal | [4] |
| Tribulus terrestris L. | Zygopyhyllaceae | Puncturevine | Microlarinus lypriformis  (Wollaston) | Curculionidae | AZ | 1963 | Kallstroemia grandiflora Torr. ex A. Gray | Zygopyhyllaceae | Minimal | [4] |
| Tribulus terrestris L. | Zygophyllaccae | Puncturevine | Microlarinus lypriformis  (Wollaston) | Curculionidae | HI | 1963 | Malva parviflora L. | Malvaceae | Minimal | [25] |
| Ulex europeaus L. | Fabaceae | Gorse | Cydia succedana  (Denis and Schiffermüller) | Tortricidae | New Zealand | 1992 | Cytisus proliferus L.f. | Fabaceae | Minimal | [54] |
| Ulex europeaus L. | Fabaceae | Gorse | Cydia succedana  (Denis and Schiffermüller) | Tortricidae | New Zealand | 1992 | Cytisus scoparius (L.) Link. | Fabaceae | Minor | [48,54,55] |
| Ulex europeaus L. | Fabaceae | Gorse | Cydia succedana  (Denis and Schiffermüller) | Tortricidae | New Zealand | 1992 | Genista lydia Boiss. | Fabaceae | Minimal | [54] |
| Ulex europeaus L. | Fabaceae | Gorse | Cydia succedana  (Denis and Schiffermüller) | Tortricidae | New Zealand | 1992 | Genista monspessulana (L.) L.A.S. Johnson | Fabaceae | Minimal | [55] |
| Ulex europeaus L. | Fabaceae | Gorse | Cydia succedana  (Denis and Schiffermüller) | Tortricidae | New Zealand | 1992 | Lotus corniculatus L. | Fabaceae | Minimal | [54] |
| Ulex europeaus L. | Fabaceae | Gorse | Cydia succedana  (Denis and Schiffermüller) | Tortricidae | New Zealand | 1992 | Lotus pedunculatus Cav. | Fabaceae | Minimal | [54] |
| Ulex europeaus L. | Fabaceae | Gorse | Cydia succedana  (Denis and Schiffermüller) | Tortricidae | New Zealand | 1992 | Lotus polyphyllus E. D. Clarke | Fabaceae | Minimal | [54] |
| Ulex europeaus L. | Fabaceae | Gorse | Cydia succedana  (Denis and Schiffermüller) | Tortricidae | New Zealand | 1992 | Lupinus arboreus Sims | Fabaceae | Minimal | [55] |
| Ulex europeaus L. | Fabaceae | Gorse | Cydia succedana  (Denis and Schiffermüller) | Tortricidae | New Zealand | 1992 | Spartium junceum L. | Fabaceae | Minimal | [54] |

USA Location codes: AZ: Arizona, CA: California, CO: Colorado, FL: Florida, HI: Hawaii, MT: Montana, NE: Nebraska, TX: Texas, UT: Utah, VA: Virginia, WY: Wyoming. NA: Not Applicable (accidental introduction)

1. Fowler SV, Syrett P, Hill RL (2000) Success and safety in the biological control of environmental weeds in New Zealand. Austral Ecol 25: 553-562. doi: 10.1046/j.1442-9993.2000.01075.x

2. Dennill GB, Donnelly D, Chown SL (1993) Expansion of host-plant range of a biocontrol agent *Trichilogaster acaciaelongifoliae* (Pteromalidae) released against the weed *Acacia longifolia* in South Africa. Agric, Ecosyst Environ 43: 1-10. doi: 10.1016/0167-8809(93)90002-7

3. Conant P (1998) A new host record for *Oidaematophorus beneficus* Yano & Heppner (Lepidoptera: Pterophoridae). Proc Hawaii Entomol Soc 33: 151-152

4. Pemberton RW (2000) Predictable risk to native plants in weed biological control. Oecologia 125: 489-494. doi: 10.1007/s004420000477

5. Takahashi M, Louda SM, Miller TEX, O'Brien CW (2009) Occurrence of *Trichosirocalus horridus* (Coleoptera: Curculionidae) on native *Cirsium altissimum* versus exotic *C. vulgare* in North American tallgrass prairie. Environ Entomol 38: 731-740. doi: 10.1603/022.038.0325

6. Wiggins GJ, Grant JF, Lambdin PL, Ranney JW, Wilkerson JB (2009) First documentation of adult *Trichosirocalus horridus* on several non-target native Cirsium species in Tennessee. Biocontrol Sci Technol 19: 993-998. doi: 10.1080/09583150903191343

7. Kok LT, Mays WT (1991) Successful biological control of plumeless thistle, *Carduus acanthoides* L. [Campanulatae: Asteraceae (= compositae)], by *Trichosirocalus horridus* (Panzer) (Coleoptera: Curculionidae) in Virginia. Biol Control 1: 197-202. doi: 10.1016/1049-9644(91)90067-A

8. McAvoy TJ, Kok LT, Mays WT (1987) Dispersal of *Trichosirocalus horridus* (Panzer) (Coleoptera: Curculionidae) in Southwest Virginia. J Entomol Sci 22: 324-329

9. Dodge G, Louda SM, Inouye D (2005) Appendices to "Colonization of thistles by biocontrol agents". Digital Repository at the University of Maryland.

10. Turner CE, Pemberton RW, Rosenthal SS (1987) Host utilization of native *Cirsium* thistles (Asteraceae) by the introduced weevil *Rhinocyllus conicus* (Coleoptera: Curculionidae) in California. Environ Entomol 16: 111-115

11. Rose KE, Louda SM, Rees M (2005) Demographic and evolutionary impacts of native and invasive insect herbivores on *Cirsium canescens*. Ecology 86: 453-465. doi: 10.1890/03-0697

12. Louda SM (1998) Population growth of *Rhinocyllus conicus* (Coleoptera: Curculionidae) on two species of native thistles in prairie. Environ Entomol 27: 834-841

13. Louda SM, Rand TA, Arnett AE, McClay AS, Shea K, et al. (2005) Evaluation of ecological risk to populations of a threatened plant from an invasive biocontrol insect. Ecol Appl 15: 234-249. doi: 10.1890/03-5212

14. Louda SM, O’Brien CW (2002) Unexpected ecological effects of distributing the exotic weevil, *Larinus planus* (F.), for the biological control of Canada thistle. Conserv Biol 16: 717-727. doi: 10.1046/j.1523-1739.2002.00541.x

15. Paynter Q, Fowler SV, Gourlay AH, Haines ML, Harman HM, et al. (2004) Safety in New Zealand weed biocontrol: a nationwide survey for impacts on non-target plants N Z Plant Prot 57: 102-107

16. Andreas JE, Schwarzländer M, De Clerck-Floate R (2008) The occurrence and potential relevance of post-release, nontarget attack by *Mogulones cruciger*, a biocontrol agent for *Cynoglossum officinale* in Canada. Biol Control 46: 304-311. doi: 10.1016/j.biocontrol.2008.05.014

17. Poinar GO, Jr., Thomas G, Prokopy RJ (1977) Micro-organisms associated with *Rhagoletis pomonella* (Tephritidae; Diptera) in Massachusetts. Proc Entomol Soc Ont 108: 19-22

18. Haines ML, Martin J-F, Emberson RM, Syrett P, Withers TM, et al. (2007) Can sibling species explain the broadening of the host range of the broom seed beetle, *Bruchidius villosus* (F.) (Coleoptera: Chrysomelidae) in New Zealand? N Z Entomol 30: 5-11

19. Syrett P, O’Donnell DJ (1987) A seed-feeding beetle for biological control of broom. Proceedings of the 40th NZ Weed Pest Control Conference: 19-22

20. Julien MH, Griffiths MW (1998) Biological control of weeds: a world catalogue of agents and their target weeds. Wallingford, U.K.: CAB International.

21. Harris P (1988) Environmental impact of weed-control insects. Bioscience 38: 542–548

22. Baker JL, Webber NAP, Johnson KK (2003) Non-target impacts of *Aphthona nigriscutis*, a biological control agent for *Euphorbia esula* (leafy spurge), on a native plant *Euphorbia robusta* In: Cullen JM, Briese DT, Kriticos DJ, Lonsdale WM, Morin L et al., editors. Proceedings of the XI International Symposium on Biological Control of Weeds, 27 April - 2 May. Canberra, Australia. pp. 247-251.

23. Willis AJ, Groves RH, Ash JE (1998) Interactions between plant competition and herbivory on the growth of *Hypericum s*pecies: a comparison of glasshouse and field results. Aust J Bot 46: 707-721. doi: 10.1071/BT97025

24. Campbell CL, McCaffrey JP (1991) Population trends, seasonal phenology, and impact of *Chrysolina quadrigemina*, *C. hyperici* (Coleoptera: Chrysomelidae), and *Agrilus hyperici* (Coleoptera: Buprestidae) associated with *Hypericum perforatum* in Northern Idaho. Environ Entomol 20: 303-315

25. Funasaki G, Lai P-Y, Nakahara LM, Beardsley J, Ota AK (1988) A review of biological control introductions in Hawaii: 1890-1985. Proc Hawaii Entomol Soc 28: 105-160

26. Andres LA. Interaction of *Chrysolina quadrigemina* and *Hypericum* spp. in California. In: Delfosse ES, editor. Proceedings of the VI International Symposium Biological Control Weeds, Vancouver, Canada, 19-25 August; 1985. Agriculture Canada, Ottawa. pp. 235-239.

27. Palmer WA, Day MD, Dhileepan K, Snow EL, Mackey AP. Analysis of the non-target attack by the lantana sap-sucking bug, *Aconophora compressa* and its implications for biological control in Australia. In: Sindel BM, Johnson SB, editors. Proceedings of the Fourteenth Australian Weeds Conference “Weed Management - Balancing People, Planet, Profit”, 6-9 September; 2004; Sydney, Australia. Weed Society of New South Wales. pp. 341-344.

28. Manners AG, Palmer WA, Burgos A, McCarthy J, Walter GH (2011) Relative host plant species use by the lantana biological control agent Aconophora compressa (Membracidae) across its native and introduced ranges. Biol Control 58: 262-270. doi: 10.1016/j.biocontrol.2011.05.013

29. Maehler MR, Ford MR (1955) *Teleonemia scrupulosa*. Proc Hawaii Entomol Soc 15: 377

30. Hight SD, Pemberton RW, Conant P, Johnson T. Attack on and use of a native Hawaiian plant by the biological control agent *Teleonemia scrupulosa* introduced against *Lantana camara* In: Cullen JM, Briese DT, Kriticos DJ, Lonsdale WM, Morin L et al., editors. Proceedings of the XI International Symposium on Biological Control of Weeds, 27 April - 2 May; 2003; Canberra, Australia. pp. 350.

31. Greathead DJ (1995) Benefits and risks of classical biological control. In: Hokkanen HMT, Lynch JM, editors. Biological Control: Benefits and Risks. Cambridge, U. K.: Cambridge University Press. pp. 53-63.

32. Davies JC, Greathead DJ (1967) Occurence of *Teleonemia scrupulosa* on *Sesamum indicum* Linn. in Uganda. Nature 213: 102-103. doi: 10.1038/213102b0

33. McFadyen REC (1998) Biological control of weeds. Annu Rev Entomol 43: 369-393. doi: 10.1146/annurev.ento.43.1.369

34. Kok LT, McAvoy TJ, Maleckij RA, Hight SD, Drea JJ, et al. (1992) Host specificity tests of *Galerucella calmariensis* (L.) and *G. pusilla* (Duft.) (Coleoptera: Chrysomelidae), potential biological control agents of purple loosestrife, *Lythrum salicaria* L. (Lythraceae). Biol Control 2: 282-290. doi: 10.1016/1049-9644(92)90020-e

35. Blossey B, Casagrande R, Tewksbury L, Landis DA, Wiedenmann RN, et al. (2001) Nontarget feeding of leaf-beetles introduced to control purple loosestrife (*Lythrum salicaria* L.). Nat Areas J 21: 368-377

36. Schooler SS, Coombs EM, McEvoy PB (2003) Nontarget effects on crepe myrtle by *Galerucella pusilla* and *G. calmariensis* (Chrysomelidae), used for biological control of purple loosestrife (*Lythrum salicaria*). Weed Sci 51: 449-455

37. Barratt BIP, Blossey B, Hokkanen HMT (2006) Post-release evaluation of non-target effects of biological control agents. In: Bigler F, Babendreier D, Kuhlmann U, editors. Environmental impact of invertebrates for biological control of arthropods: methods and risk assessment. Wallingford, U. K.: CABI Publishing. pp. 166-186.

38. Forno IW, Fichera J, Prior S. Assessing the risk to *Neptunia oleracea* Lour. by the moth, *Neurostrota gunniella* (Busck), a biological control agent for *Mimosa pigra* L. In: Spencer NR, editor. Proceedings of the X International Symposium on Biological Control of Weeds 4-14 July; 2000; Montana State University, Bozeman, Montana, USA. pp. 449-457

39. Willis AJ, Kilby MJ, McMaster K, Cullen JM, Groves RH (2003) Predictability and acceptability: potential for damage to nontarget native plant species by biological control agents for weeds. In: Spafford-Jacob H, Briese DT, editors. Improving the Selection, Testing and Evaluation of Weed Biological Control Agents. Glen Osmond, South Australia CRC for Australian Weed Management Technical Series.

40. Taylor DBJ, Heard TA, Paynter Q, Spafford H (2007) Nontarget effects of a weed biological control agent on a native plant in Northern Australia. Biol Control 42: 25-33. doi: 10.1016/j.biocontrol.2007.04.002

41. Pemberton RW, Liu H (2007) Control and persistence of native Opuntia on Nevis and St. Kitts 50 years after the introduction of *Cactoblastis cactorum*. Biol Control 41: 272-282. doi: 10.1016/j.biocontrol.2007.02.002

42. Pemberton RW, Cordo H (2001) Potential and risk of biological control of *Cactoblastis cactorum* (Lepidoptera: Pyralidae) in North America. Fla Entomol 84: 513-526

43. Jezorek HA, Stiling PD, Carpenter JE (2010) Targets of an invasive species: Oviposition preference and larval performance of *Cactoblastis cactorum* (Lepidoptera: Pyralidae) on 14 North American opuntioid cacti. Environ Entomol 39: 1884-1892. doi: 10.1603/en10022

44. Zimmermann HG, Moran VC, Hoffmann JH (2000) The renowned cactus moth, *Cactoblastis cactorum*: its natural history and threat to native *Opuntia* floras in Mexico and the United States of America. Divers Distrib 6: 259-269. doi: 10.1046/j.1472-4642.2000.00088.x

45. Pemberton RW (1995) *Cactoblastis cactorum* (Lepidoptera: Pyralidae) in the United States: an immigrant biological control agent or an introduction of the nursery industry? Am Entomol 41: 230-232

46. Zimmermann H, Bloem S, Klein H (2004) Biology, history, threat, surveillance and control of the cactus moth, *Cactoblastis cactorum*. Vienna, Austria: FAO. 40 p.

47. Diehl JW, McEvoy PB. Impact of the cinnabar moth (*Tyria jacobaeae*) on *Senecio triangularis*, a non-target native plant in Oregon. In: Delfosse ES, editor. Proceedings of the VII International Symposium on Biological Control of Weeds, 6-11 March; 1988; Rome, Italy. pp. 119-126.

48. Fowler SV, Gourlay AH, Hill RH, Withers T. Safety in New Zealand weed biocontrol: a retrospective analysis of host-specificity testing and the predictability of impacts on non-target plants. In: Cullen JM, Briese DT, Kriticos DJ, Lonsdale WM, Morin L et al., editors. Proceedings of the XI International Symposium on Biological Control of Weeds, 27 April - 2 May; 2004; Canberra, Australia. pp. 265-270.

49. Bucher GE, Harris P (1961) Food-plant spectrum and elimination of disease of cinnabar moth larvae, *Hypocrita jacobaeae* (L.) (Lepidoptera: Arctiidae). Can Entomol 93: 931-936. doi: 10.4039/Ent93931-10

50. Parker HL (1960) Starvation tests with larvae of the cinnabar moth. J Econ Entomol 53: 472-473

51. Helson GAH (1974) Beneficial insects. Cinnabar moth and ragwort seed fly. N Z J Agric 128 56-59

52. Moran PJ, DeLoach CJ, Dudley TL, Sanabria J (2009) Open field host selection and behavior by tamarisk beetles (*Diorhabda* spp.) (Coleoptera: Chrysomelidae) in biological control of exotic saltcedars (*Tamarix* spp.) and risks to non-target athel (*T. aphylla*) and native *Frankenia* spp. Biol Control 50: 243-261. doi: 10.1016/j.biocontrol.2009.04.011

53. Dudley TL, Kazmer DJ (2005) Field assessment of the risk posed by *Diorhabda elongata*, a biocontrol agent for control of saltcedar (*Tamarix* spp.), to a nontarget plant, *Frankenia salina*. Biol Control 35: 265-272. doi: 10.1016/j.biocontrol.2005.05.002

54. Paynter Q, Gourlay AH, Oboyski PT, Fowler SV, Hill RL, et al. (2008) Why did specificity testing fail to predict the field host-range of the gorse pod moth in New Zealand? Biol Control 46: 453-462. doi: 10.1016/j.biocontrol.2008.05.004

55. Withers TM, Hill RL, Paynter Q, Fowler SV, Gourlay A (2008) Post-release investigations into the field host range of the gorse pod moth *Cydia succedana* Denis & Schiffermüller (Lepidoptera: Tortricidae) in New Zealand. N Z Entomol 31: 67-76. doi: 10.1080/00779962.2008.9722168
